# Clinical and immunological outcomes after randomized trial of baked milk oral immunotherapy for milk allergy

**DOI:** 10.1172/jci.insight.184301

**Published:** 2025-01-09

**Authors:** Jennifer A. Dantzer, Sloan A. Lewis, Kevin J. Psoter, Aaron Sutherland, April Frazier, Eve Richardson, Synaida Maiche, Gregory Seumois, Bjoern Peters, Robert A. Wood

**Affiliations:** 1 Division of Pediatric Allergy, Immunology, and Rheumatology, Department of Pediatrics, John Hopkins University School of Medicine, Baltimore, Maryland, USA.; 2 La Jolla Institute for Immunology, La Jolla, California, USA.; 3 Division of General Pediatrics, Department of Pediatrics, Johns Hopkins University School of Medicine, Baltimore, Maryland, USA.; 4 Department of Medicine, University of California San Diego, La Jolla, California, USA.

**Keywords:** Clinical trials, Immunology, Allergy, Cellular immune response, Immunotherapy

## Abstract

**BACKGROUND:**

Cow’s milk (CM) allergy is the most common food allergy in young children. Treatment with oral immunotherapy (OIT) has shown efficacy, but high rates of adverse reactions. The aim of this study was to determine whether baked milk OIT (BMOIT) could reduce adverse reactions while still inducing desensitization, and to identify immunological correlates of successful BMOIT.

**METHODS:**

This phase II, randomized trial evaluated the safety and efficacy of BMOIT in milk-allergic children 3–18 years old. After the initial placebo-controlled first year of treatment, placebo-treated participants crossed over to active BMOIT. Double-blind, placebo-controlled oral food challenges (OFCs) were conducted with BM after year 1 and to both BM and unheated milk (UM) after year 2. IgG and IgE antibodies were measured along with CM-specific (CM^+^) CD4^+^ memory T cell populations, profiled using flow cytometry and scRNA-Seq.

**RESULTS:**

Twenty-one of 30 (70%) reached the primary endpoint of tolerating 4044 mg of BM protein at month 24, and 11 of 30 tolerated 2000 mg or more of UM protein. Dosing symptoms were common, but more than 98% were mild, with no severe reactions. Immunological changes associated with desensitization included increased CM IgG4, CM^+^ *FOXP3*^+^ cells, and Tregs and corresponding decreases in CM IgE, CM^+^ Th2A cells, and CD154^+^ cells. T cell and antibody measurements were combined to build a model that predicted UM OFC outcomes.

**CONCLUSION:**

BMOIT was well tolerated and induced desensitization to BM and UM. This desensitization corresponded to redistribution within antigen-specific antibody and T cell compartments that provided insight into the mechanistic changes that occur with OIT treatment.

**TRIAL REGISTRATION:**

ClinicalTrials.gov NCT03462030.

**FUNDING: Myra Reinhardt Family Foundation (grant number 128388), NIH/NIAID (U19AI135731, T32AI125179, S10OD025052):**

## Abstract 2

Baked milk oral immunotherapy induced desensitization to baked and unheated milk, which corresponded to redistribution within antigen specific antibody and T cell compartments.

## Graphical abstract

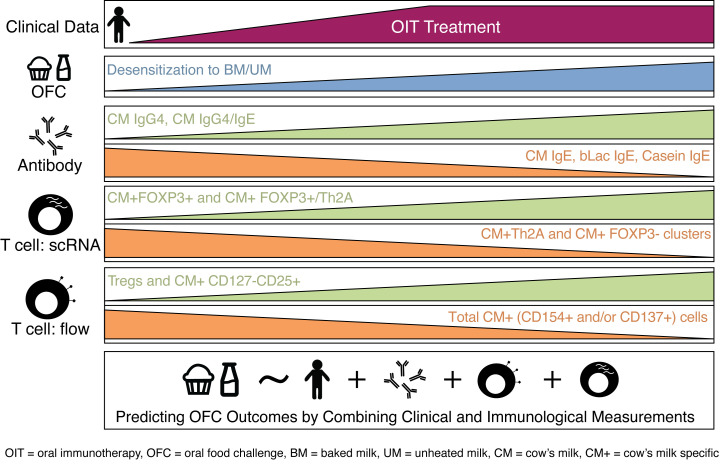

## Introduction

Cow’s milk (CM) allergy (CMA) is the most common food allergy among children (1). Many children outgrow CMA, but for some it will persist into adolescence and adulthood (2, 3). Prior studies have found that those reacting to less than 10 mL of milk and those with a history of a reaction to baked milk (BM) are at increased risk for persistence (4–6). Because of the ubiquitous nature of dairy, CM avoidance can be very difficult, with the risk of frequent and often severe reactions. In addition, CMA can lead to increased healthcare utilization, financial burden, psychosocial burden, and nutritional deficits, especially for those who cannot tolerate BM (7, 8). For all these reasons, a safe and effective treatment of CMA is greatly needed, especially for those with a more severe phenotype who are unlikely to naturally outgrow their allergy.

Oral immunotherapy (OIT) for peanut was the first FDA-approved treatment for food allergy, with promising results for additional foods (9, 10). OIT involves the daily ingestion of escalating doses of allergen with the goal of inducing desensitization (a loss of clinical reactivity with regular consumption of the allergen) (10, 11). Approximately 80% who undergo food OIT achieve desensitization, but prior studies have shown high rates of adverse reactions leading to 10%–25% of participants dropping out of OIT trials (12). For CMA specifically, prior studies have shown that milk OIT can be particularly problematic, with increased risk for severe reactions and OIT failure (13, 14). A recent meta-analysis showed increased risk of epinephrine use (relative risk [RR]: 8.45, 95% CI: 2.02–35.27) with unheated milk (UM) OIT compared with avoidance (15). This prompts the need for alternative approaches that enhance safety while maintaining or improving efficacy.

Studies have shown that extensive heating of milk proteins, such as through baking, leads to conformational changes in proteins that makes them less allergenic; yet, they maintain the antigenic properties needed to induce tolerance (16, 17). For individuals with CMA, 50% to 70% will tolerate extensively BM, such as muffins (16). Those who can tolerate BM have less severe reactions to liquid milk, typically gain nonreactivity to less-cooked forms, and have a high rate of outgrowing CMA in the next few years (16, 18).

BM introduction has become a routine part of clinical practice; however, this is not an option for those patients with a more severe milk allergy and who are less likely to outgrown their CMA. Therefore, we initiated a study of BM oral immunotherapy (BMOIT) that utilized lower starting doses than those typically used in clinical practice or prior research, in addition to more gradual dose escalations (19).

Despite the now extensive research on OIT, the immune mechanisms of desensitization remain poorly understood (20). Serologic changes, especially in allergen-specific IgG4, have been shown in most studies, as have changes in basophil reactivity (21). Allergen-specific T cell populations, including Th2 cells and Tregs, are known to play roles in food allergy disease and the induction of tolerance (22, 23), but the role of T cells in OIT outcomes remains unclear. This can be attributed to consistency of findings, which is in part due to the difficulty of isolating/measuring these rare populations ex vivo (22, 24). There is also a lack of analyses performed with a hypothesis-driven standard that produce high-confidence findings. This clinical trial provided the opportunity to address this gap in knowledge and address several key predictive/mechanistic questions: (a) Can we predict whether a patient received treatment based on immunological measures in a blinded fashion? (b) What are the immunological measures that change over the course of OIT? (c) Can we use immunological measures to predict desensitization?

In this phase II, single-center, randomized, double-blind placebo-controlled (DBPC) study of BMOIT in children with severe CMA, our primary objective was to understand the safety and efficacy of BMOIT, with the secondary objective of exploring immunological correlates of desensitization. Here, we present the clinical outcomes of the open-label second year of treatment as well as immunological studies over the entire 2-year study period.

## Results

### Description of clinical trial study population.

**Figure 1:**
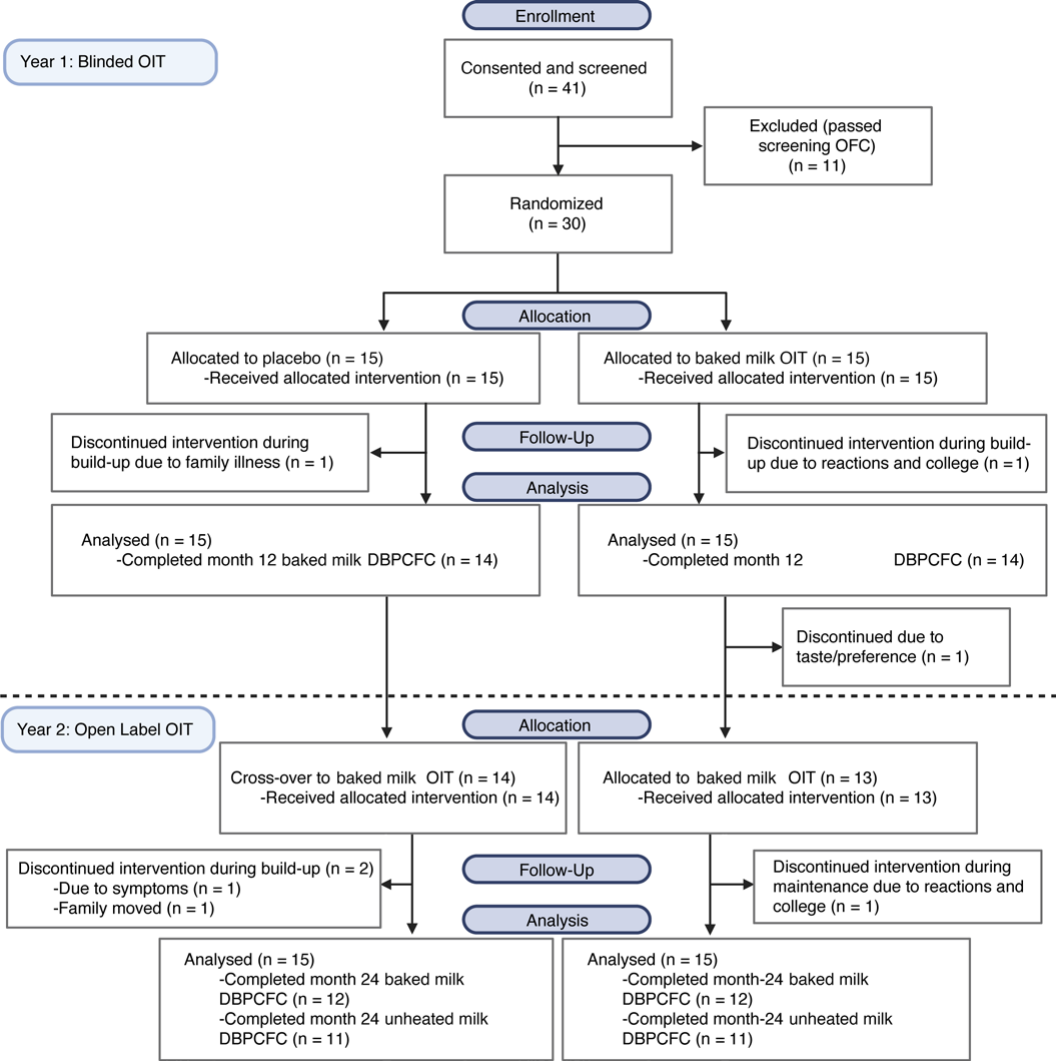
CONSORT diagram. Flowchart of participants’ disposition throughout the study.

**Figure 2:**
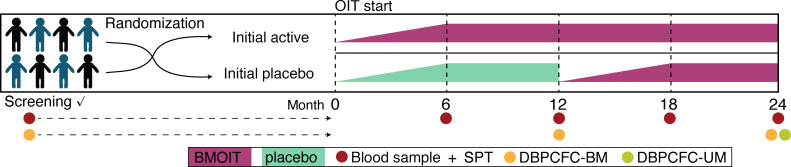
Trial design and time points for outcomes. Timeline showing baked milk (BMOIT) and placebo oral immunotherapy (OIT) groups and timing of blood draw (mechanistic studies), skin prick tests (SPT), and double-blind placebo-controlled food challenge (DBPCFC) to baked (BM) and unheated (UM) milk.

We enrolled 41 patients with CMA from March 2018 to October 2019 (Figure 1), as detailed previously (25). Eleven were ineligible, as they failed to react during their screening DBPC food challenge (DBPCFC) to 444 mg (<1 tablespoon) of BM (26). Thirty patients met all eligibility criteria and were randomized 1:1 to BM or placebo OIT during the first year of treatment (Figure 1 and Figure 2). Twenty-eight participants completed the 12-month BM DBPCFC. All were then invited to receive an additional 12 months of open-label BMOIT. Twenty-seven started open-label BMOIT, with 24 completing the month 24 (end of treatment) BM DBPCFC and 22 completing the month 24 UM DBPCFC. The last participant completed their final DBPCFC in February 2022.

Demographic and other baseline characteristics of the 30 randomized participants are summarized in Supplemental Table 1 (supplemental material available online with this article; https://doi.org/10.1172/jci.insight.184301DS1) and were previously published (25). Sixteen (53%) were male and 14 (47%) were female. The median age at enrollment was 11 years (range: 3–18 years). All had a prior reaction history to some form of milk (63% UM and BM, 27% UM only, and 10% BM only). Twenty-six (87%) had multiple food allergies. There was high prevalence of other atopic diseases at enrollment, with 53% having atopic dermatitis, 67% asthma, and 70% allergic rhinitis.

During year 1, all 30 randomized participants completed the initial dose escalation (IDE) and tolerated the required minimum dose of 3 mg of BM/placebo. Participants then began daily OIT home dosing. Two participants withdrew during buildup in year 1 (related to starting college and family reasons) and hence 28 of 30 (93%) underwent the 12-month BM DBPCFC (25). After the 12-month DBPCFC, the groups were unblinded and all participants were offered open-label BMOIT for an additional 1 year. One participant in the active group withdrew after the 12-month BBPCFC, prior to starting year 2 due to taste/not wanting to eat more muffins; therefore, 27 participants received open-label BMOIT during the second year of treatment (14 initial placebo group, 13 initial active group). In year 2, two participants withdrew from the placebo crossover group (1 due to GI symptoms, 1 due to moving) and 1 withdrew from the initial active dose group due to symptoms with dosing and starting college.

Twenty-four participants (12 of each group) completed the end of treatment, month 24 BM DBPCFC. One participant in each group (*n* = 2) did not tolerate 2000 mg of BM and were not eligible for the UM DBPCFC.

### Clinical trial efficacy outcomes.

**Figure 3:**
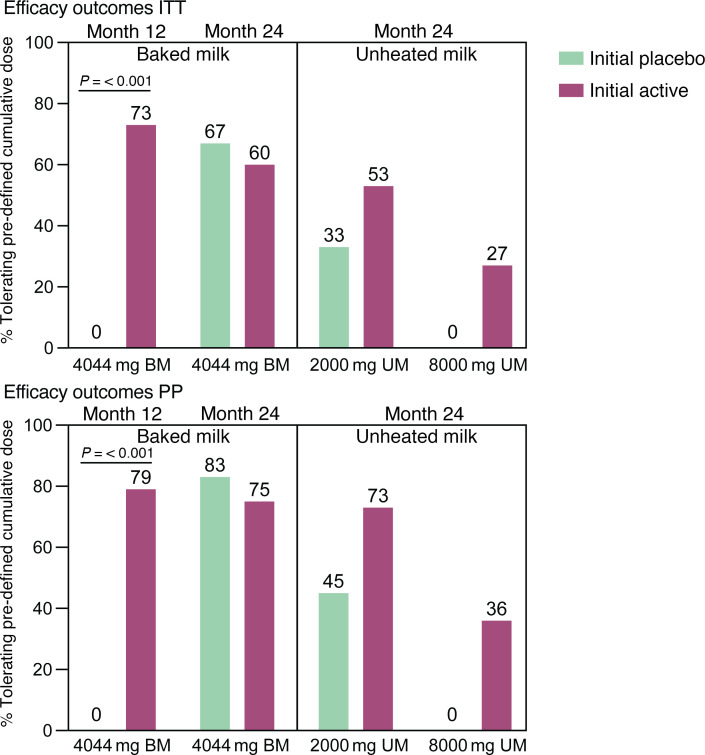
Efficacy outcomes. Percentage of each group tolerating predefined total cumulative dose of baked milk (months 12 and 24) and unheated milk (month 24) protein by treatment group for intent to treat (ITT) and per-protocol (PP) populations. There was a significant difference (*P* < 0.05) between groups only at month 12. For the ITT analysis, *n* = 30 (month 12 and month 24). For the PP analysis, *n* = 28 (month 12 baked), *n* = 24 (month 24 baked), and *n* = 22 (month 24 unheated). Statistical analyses were performed using Fisher’s exact test.

At baseline, the median maximum tolerated dose (MTD) was 44 mg of BM protein (approximately one-quarter teaspoon) in the active group and 144 mg (approximately 1 teaspoon) in the placebo group. At month 12 and month 24, participants underwent a DBPCFC to up to a cumulative dose of 4044 mg of BM protein (approximately a half cup). In the month 12 BM DBPCFC, 11 of 15 (73%) in the active group compared with 0 of 15 in the placebo group tolerated 4044 mg of BM (*P* < 0.001) (Figure 3) (25). In the month 24 BM DBPCFC, 9 of 15 (60%) of the initial active group and 10 of 15 (67%) of the placebo crossover group tolerated 4044 mg of BM, with no significant difference between groups (Figure 3 and Supplemental Table 2). In the per-protocol (PP) analysis, the majority of participants in both groups tolerated 4044 mg of BM (9 of 12 [75%]) in the initial active group compared with 10 of 12 (83%) in the initial placebo group. Coincidently, when we collapsed the initial treatment groups and analyzed the PP population based on time on treatment (0, 12, and 24 months), we found that 21 of 26 (81%) tolerated 4044 mg of BM after 12 months of active BMOIT (Supplemental Figure 1). This was significant compared with the baseline, as was the comparison of 24 months to baseline for the individuals in the initial active BMOIT group (Supplemental Figure 1).

Twenty-two participants, 11 in each group, underwent the 24-month UM DBPCFC, with a possible maximum cumulative dose of 8030 mg of milk protein (approximately 1 cup). Eight of 15 (53%) in the initial active group compared with 5 of 15 (33%) in the placebo crossover group tolerated 2000 mg or more cumulative UM protein (*P* = 0.46), while 4 of 15 (27%) versus 0 of 15, respectively, tolerated the maximum cumulative dose of 8030 mg of UM protein (*P* = 0.1) (Figure 3). In the PP analysis, in the initial active group, 8 of 11 (73%) tolerated 2000 mg or more cumulative UM protein and 4 of 11 (36%) tolerated 8000 mg compared with 5 of 11 (45%) and 0 of 11 in the initial placebo group, respectively (*P* = 0.39 and 0.0) (Figure 3).

**Figure 4:**
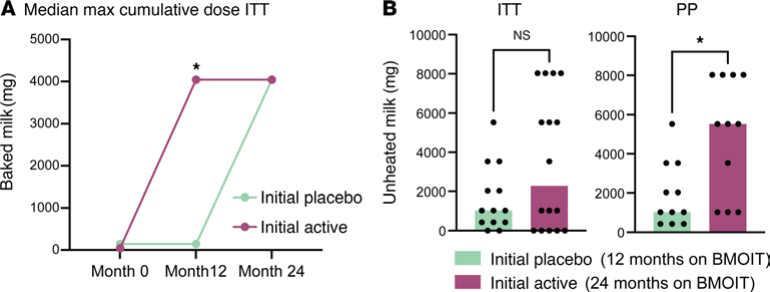
Maximum cumulative tolerated dose. (**A**) Median value of the maximum cumulative tolerated dose of baked milk at baseline, month 12, and month 24 split by initial treatment group in the intent-to-treat (ITT) population (*n* = 30). (**B**) Maximum tolerated dose of unheated milk at month 24 for each participant (dots) split by initial treatment group. Group medians are indicated by the bars. Left panel is ITT population (*n* = 30). Right is per-protocol (PP) analysis (*n* = 22). Statistical analyses were performed using Mann-Whitney *U* tests. *P < 0.05.

We also evaluated the MTD of BM and UM (Figure 4A). At baseline, the median MTD of BM was 44 mg of CM protein (range: 4–144 mg) in the initial active group compared with 144 mg (range: 4–144 mg) in the initial placebo group (*P* = 0.25). At month 12, the initial active group had a median change in BM MTD from a baseline of 3900 mg compared with 0 in the placebo group (*P* = 0.0001) (25). At month 24, both groups had a median change in BM MTD from a baseline of 3900 mg, with no difference between groups (12 vs. 24 months of treatment) (Figure 4A). In those who underwent the 24-month UM DBPCFC, the median MTD of UM was 2780 mg, with a range of 430 mg to 8030 mg (Figures 4B and Supplemental Figure 2). There was a significant difference in the median MTD of UM protein after 24 versus 12 months of treatment based on the PP analysis (median MTD of 5530 mg initial active group vs. 1030 mg in the initial placebo group, *P* = 0.01), but this was not statistically significant in the intention-to-treat (ITT) analysis (3530 mg vs. 1030 mg, *P* = 0.11) (Figure 4B).

In addition, we reviewed the severity of symptoms during the 24-month DBPCFC to both BM and UM. Severity of symptoms during the DBPCFC to BM and UM was determined based on the CoFAR Grading Scale for Systemic Allergic Reactions version 3.0 (27), which has 5 levels of increasing severity, ranging from mild symptoms involving 1 organ system (Grade 1) to death (Grade 5). Five of 24 participants who underwent the 24-month BM oral food challenge (OFC) had a reaction with 2 Grade 1 reactions and 3 Grade 2 reactions (all Grade 2 due to mild symptoms in 2 organ systems). Eighteen of 22 participants had a reaction during the UM OFC. There were 6 Grade 1 reactions, 9 Grade 2 reactions, and 3 Grade 3 reactions. There were no Grade 4 or Grade 5 reactions in any of the OFCs.

Overall, these analyses of food challenge outcomes at the 24-month time point showed that BM OIT appears to be effective at desensitizing patients to both BM and some amount of UM. There were some trends, but no statistically significant differences for greater efficacy in the initial active group over the initial placebo group (24 vs. 12 months of treatment).

### Clinical trial adverse events.

Safety and adverse events (AEs) were assessed throughout the trial. Safety data from the first year of blinded treatment was previously reported (25). During the open-label period, 9 of 13 in the initial active group and 14 of 14 in the placebo crossover group had at least 1 AE (incidence rate ratio [IRR]: 0.69, 95% CI: 0.26–1.72) (Supplemental Table 3). The average number of AEs per person during the open-label treatment year was 66 in the initial active group (range: 0–379) and 19 in the placebo crossover group (range: 3–68).

Overall, during open-label treatment, dosing-related reactions were common, but typically mild. There were symptoms with 1043 of 8914 (12%) BMOIT doses (Table 1). Eight of 13 in the initial active group compared with 14 of 14 in the placebo crossover group had at least 1 dosing-related AE in year 2 (IRR: 0.62, 95% CI: 0.22–1.57) (Supplemental Table 3). Greater than 98% of dosing-related reactions were mild and there were no severe reactions. When considering all participants, 20 of 27 (74%) had any AE with the highest severity of mild and 26% had AEs with the highest severity of moderate. The most common symptoms were oropharyngeal (OP) and GI (Table 1). The initial active group reported more OP symptoms (IRR: 8.03, 95% CI: 6.40–10.15) and GI symptoms (IRR: 1.82, 95% CI: 1.36–2.45) than the placebo crossover group in year 2. No significant difference in skin or lower respiratory tract symptoms was found between groups.

For the initial active group, the number of dosing-related reactions decreased over time, with fewer dosing-related reactions in year 2 compared with year 1, both overall (18.6% vs. 42% of doses; IRR: 0.44, 95% CI: 0.41–0.48) and in the maintenance phase (18.6% vs. 37% of doses; IRR: 0.50, 95% CI: 0.46–0.55) (Supplemental Table 4). Although OP and GI remained the most common, both significantly decreased compared with year 1 (OP-IRR: 0.54, 95% CI: 0.49–0.59; GI-IRR: 0.19, 95% CI: 0.16–0.23).

Within the placebo crossover group, there was a higher rate of dosing-related AEs during buildup compared with maintenance (IRR: 2.74, 95% CI: 1.9–4.0; median 10.5 AEs per person in buildup compared with 3 in maintenance) and more doses that required any medication during buildup (IRR: 2.21, 95% CI: 1.18–4.44) (Table 1).

Approximately 1% of doses (102 of 8914) required treatment for a dosing-related reaction, with the most common treatment being antihistamines. Compared with the placebo crossover group, the initial active group required fewer doses to be treated with any medication (IRR: 0.57, 95% CI: 0.37–0.87), antihistamines (IRR: 0.58, 95% CI: 0.37–0.92), or oral steroids (IRR: 0.13, 95% CI: 0.003–0.94), with no significant difference in use of albuterol or epinephrine. Epinephrine was used for 1 dosing-related reaction and a total of 4 times (3 during buildup and once in maintenance) by 3 individuals, all in the placebo crossover group (Supplemental Table 5).

Overall, we found that dosing-related reactions were common, with the most common symptoms being OP and GI; however, more than 98% of reactions were mild, and reactions appeared to decrease over time.

### Measurement of immunologic outcomes longitudinally across clinical trial.

Antigen-specific antibodies and CD4^+^ memory T cells were measured longitudinally throughout the trial (Figure 2). We sought to determine which immunologic measurements, if any, were associated with clinical outcomes. Data on skin tests and serologic biomarkers in year 1 have been previously reported (25). Here, we describe those measures for year 2 as well as detailed cellular immune studies from month 0 through month 24.

### Selection of antigen-specific CD4^+^ T cell populations from flow cytometry and scRNA-Seq datasets to predict BMOIT treatment versus placebo while blinded for treatment groups.

**Figure 5:**
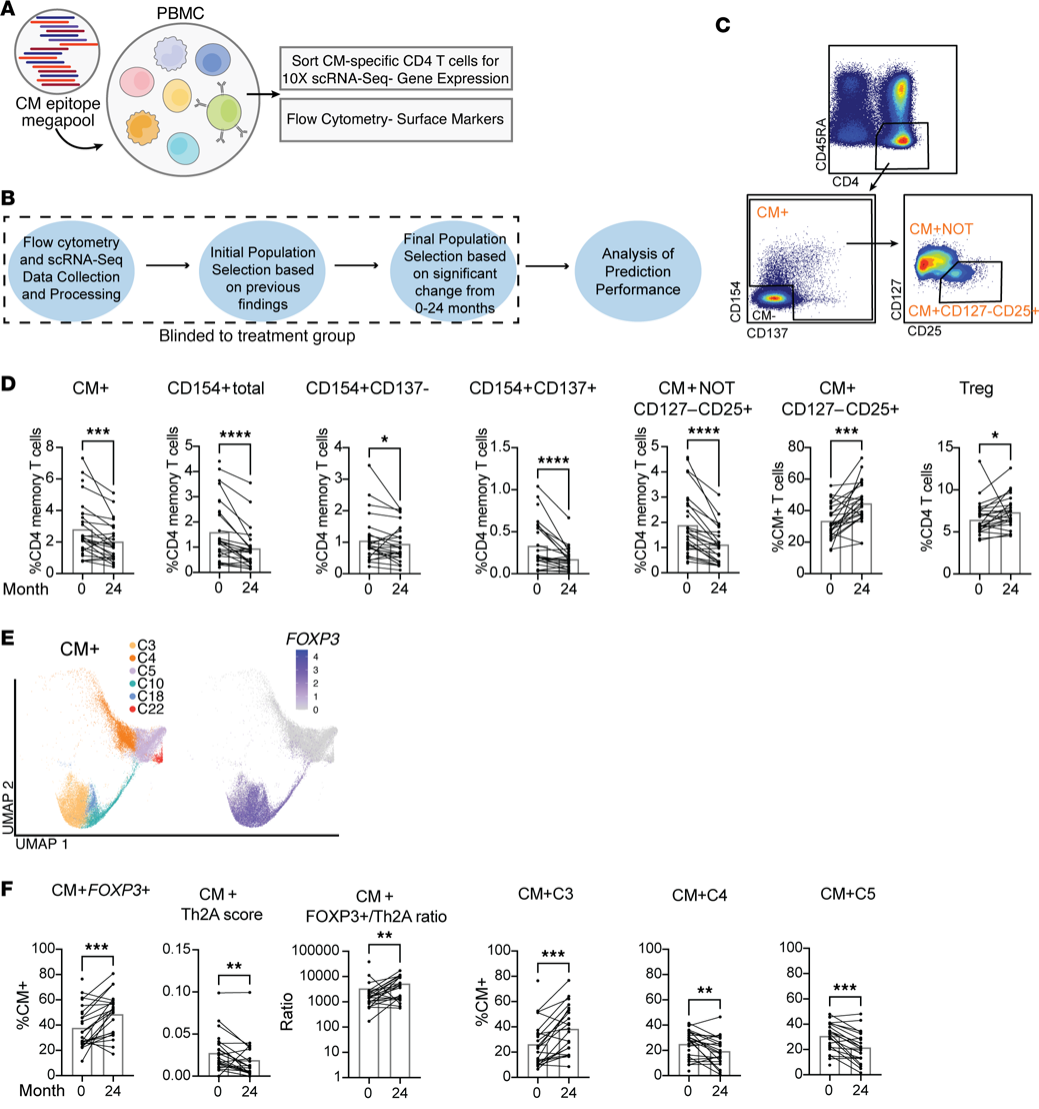
Blinded selection of CD4^+^ T cell populations from flow cytometry and scRNA-Seq assays for prediction of BMOIT treatment group. (**A**) Graphical representation of assay to identify/isolate CM-specific T cells. (**B**) Description of population selection analysis steps. (**C**) Flow cytometry populations selected for blinded analysis. Flow plot example of CD4^+^ memory CM^+^ populations. (**D**) Bar plots showing paired analysis of each selected population from month 0 to 24. (**E**) scRNA-Seq populations selected for blinded analysis. UMAP plots of CM^+^ clusters and *FOXP3* expression in those clusters. (**F**) Bar plots showing paired analysis of each selected population from month 0 to 24. We performed this assay on PBMCs from 28 participants. Statistical analyses were performed using paired Wilcoxon’s tests. *P < 0.05, **P < 0.01, ***P < 0.001, ****P < 0.0001.

To examine the mechanisms of tolerance during BMOIT, we leveraged our previously published method (28) of isolating CM-specific CD4^+^ memory T cells expressing CD154 and/or CD137 (CM^+^). Briefly, we stimulated total PBMCs with a pool of T cell–reactive CM peptides for 6 hours followed by flow cytometry to examine surface markers and further sorting of CM^+^ memory CD4^+^ T cells (CD154^+^ and/or CD137^+^) for 10× Genomics scRNA-Seq (Figure 5A). We additionally sorted CM^–^ (CD154^–^CD137^–^) cells from each sample as a control. We performed this assay on PBMCs from the 28 individuals longitudinally (5 time points; not all 28 individuals had PBMCs at every time point) across the BMOIT clinical trial (Figure 2). We included DMSO controls to show significant induction of the CM^+^ cells over background (Supplemental Figure 3, C and D).

Given the high dimensionality of our dataset, it is important to avoid overfitting due to multiple hypothesis testing. Therefore, we focused our analysis on (a) predefined T cell populations that we had previously identified as modulated in a cross-sectional study of allergic versus non-allergic individuals (28), (b) populations that have been associated with tolerance, and (c) CM^+^ T cell populations and the scRNA-Seq clusters they are derived from (Supplemental Table 6). The initial analysis was performed in a blinded fashion to the initial treatment group of each individual. We checked each cell population for significant changes between baseline (month 0) (no individuals on treatment) and month 24 (all individuals on treatment) and only moved forward with those that had a significant change in either direction (Figure 2, Figure 5B, and Supplemental Table 6).

For the flow cytometry analysis, 7 predefined populations showed significant differences with BMOIT (Figure 5, C and D, Supplemental Table 6, and Supplemental Figure 3). These included CM^+^ memory CD4^+^ populations defined by expression of CD154 and CD137 (CM^+^, total CD154^+^, CD154^+^CD137^–^, and CD154^+^CD137^+^), which all decreased significantly from month 0 to 24 (Figure 5D). The gating for these populations was set to replicate the 10× sorted data, so are less strict and may include non–antigen-specific cells. In Supplemental Figure 3, we include a strict gating strategy to show percentages for these gates and show that these populations do not outperform the less strict gates in predicting outcomes (Supplemental Figure 3, E–G). We additionally included CM^+^ CD127^–^CD25^+^ cells, which we previously identified to be increased in CMA versus non-CMA individuals using the same methods (28). Interestingly, this population increased from month 0 to 24 (*P* = 0.0002; Figure 5D). As total CM^+^ cells decreased over the time period, we included a not-gated (NOT) sample from the CM^+^ CD127^–^CD25^+^ population and back-calculated the percentage of total CD4^+^ memory cells (Figure 5D). Non–antigen-specific Tregs were also gated by CD4^+^CD127^–^CD25^+^, as these cells have been previously associated with tolerance. These cells increased significantly from 0 to 24 months (*P* = 0.0104; Figure 5D).

The analysis of the scRNA-Seq dataset was performed following the same processing steps we previously described (28) and detailed in the Methods. Briefly, clustering was performed on CM^+^ and CM^–^ sorted fractions together to determine overlap, and true CM^+^ cells were defined as those falling into clusters that were made up of more than 80% CM^+^ sorted cells. This analysis revealed 6 scRNA-Seq clusters with distinct gene expression profiles (Figure 5, E and F, Supplemental Table 6, and Supplemental Figure 4, A–E). Three of the CM^+^ clusters showed significant increases or decreases from 0 to 24 months (C3, C4, C5). CM^+^ cluster C3 was *FOXP3*^+^ MHCII markers and increased over time (*P* = 0.002). Clusters C4 and C5 were both *FOXP3*^–^, were distinguished by *CCR7^+^* (C4) and Th1/Th17 marker expression (C5), and decreased over time (*P* = 0.009 and *P* = 0.0001, respectively; Figure 5, E and F, and Supplemental Figure 4E). CM^+^ cells expressing *FOXP3* (CM^+^ *FOXP3*^+^) were included based on our previous findings and significantly increased from 0 to 24 months (*P* = 0.0009; Figure 5, E and F). CM^+^ cells expressing pathogenic Th2/Tfh markers (CM^+^ Th2A: *GATA3*, *IL4*, *IL5*, *IL13*, *IL9*, *CCL1*, *IL3*, *IL13*, *PLAC8*, *CSF2*, *HPGDS*, *CRLF2*, *PPARG*, *IL1RL1*, *PTGS2*, *IL17RB*, *KLRB1*, *HDC*, and *H2AFZ*) previously shown to be associated with CMA (28) were included and significantly decreased over time (*P* = 0.008; Figure 5F and Supplemental Figure 4F). The CM^+^ *FOXP3*^+^/Th2A cell ratio was calculated and showed a significant increase from 0 to 24 months (*P* = 0.003; Figure 5F). Detailed descriptions of all populations from the flow cytometry and scRNA-Seq analyses are listed in Supplemental Table 6.

### CM^+^ CD4^+^ T cell populations defined by scRNA-Seq best determine BMOIT treatment group.

**Figure 6:**
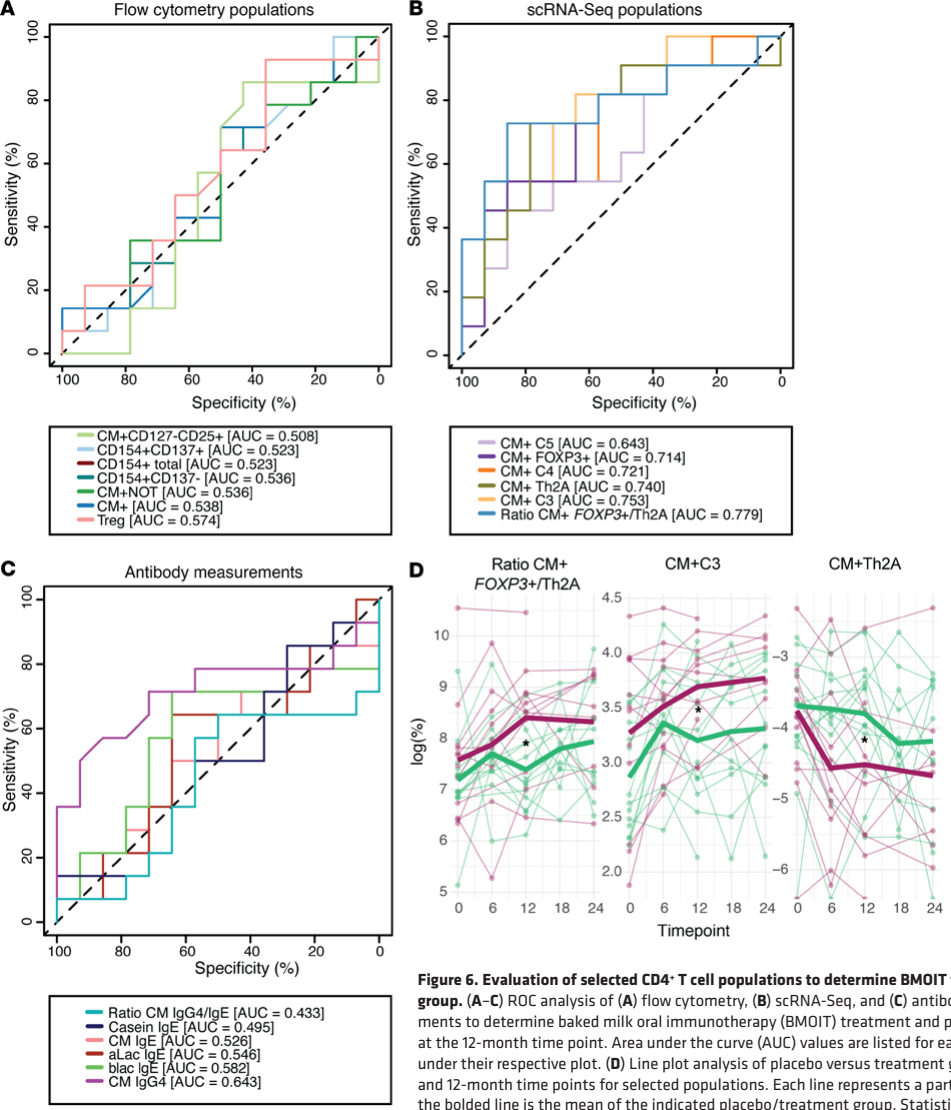
Evaluation of selected CD4^+^ T cell populations to determine BMOIT treatment group. (**A**–**C**) ROC analysis of (**A**) flow cytometry, (**B**) scRNA-Seq, and (**C**) antibody measurements to determine baked milk oral immunotherapy (BMOIT) treatment and placebo groups at the 12-month time point. Area under the curve (AUC) values are listed for each population under their respective plot. (**D**) Line plot analysis of placebo versus treatment group at 0-, 6-, and 12-month time points for selected populations. Each line represents a participant and the bolded line is the mean of the indicated placebo/treatment group. Statistical analyses were performed using Mann-Whitney *U* tests (*n* = 28). *P < 0.05.

With the final 7 flow cytometry and 6 scRNA-Seq T cell populations selected, we were unblinded to the groups to evaluate how well the selected populations could differentiate initial active BMOIT versus initial placebo after the first 12 months. We evaluated the performance by ROC analysis using the percentage of each flow cytometry (Figure 6A) or scRNA-Seq (Figure 6B) population per individual at the 12-month time point. None of the populations defined by flow cytometry had significant predictive power (AUC < 0.6; Figure 6A). In contrast, the scRNA-Seq populations had several predictors with AUC greater than 0.6, with the best being the CM^+^ *FOXP3*^+^/Th2A ratio (AUC = 0.779; Figure 6B). Importantly, this measure outperformed the predictive power of antibody measurements taken at the same time point, where CM IgG4 performed best (AUC = 0.643; Figure 6C). The populations that performed best in the ROC analysis (CM^+^ *FOXP3*^+^/Th2A, CM^+^ C3, CM^+^ Th2A) were also significantly different in a direct comparison between placebo and treatment groups at the 12-month time point, where higher percentages of CM^+^ *FOXP3*^+^/Th2A and CM^+^ C3, and lower percentages of CM^+^ Th2A cells distinguished initial active versus initial placebo (Figure 6D). No other time point comparisons for any population reached significance (Supplemental Figure 5).

### CM^+^ CD4^+^ T cell populations redistribute with 12 months of BMOIT.

**Figure 7:**
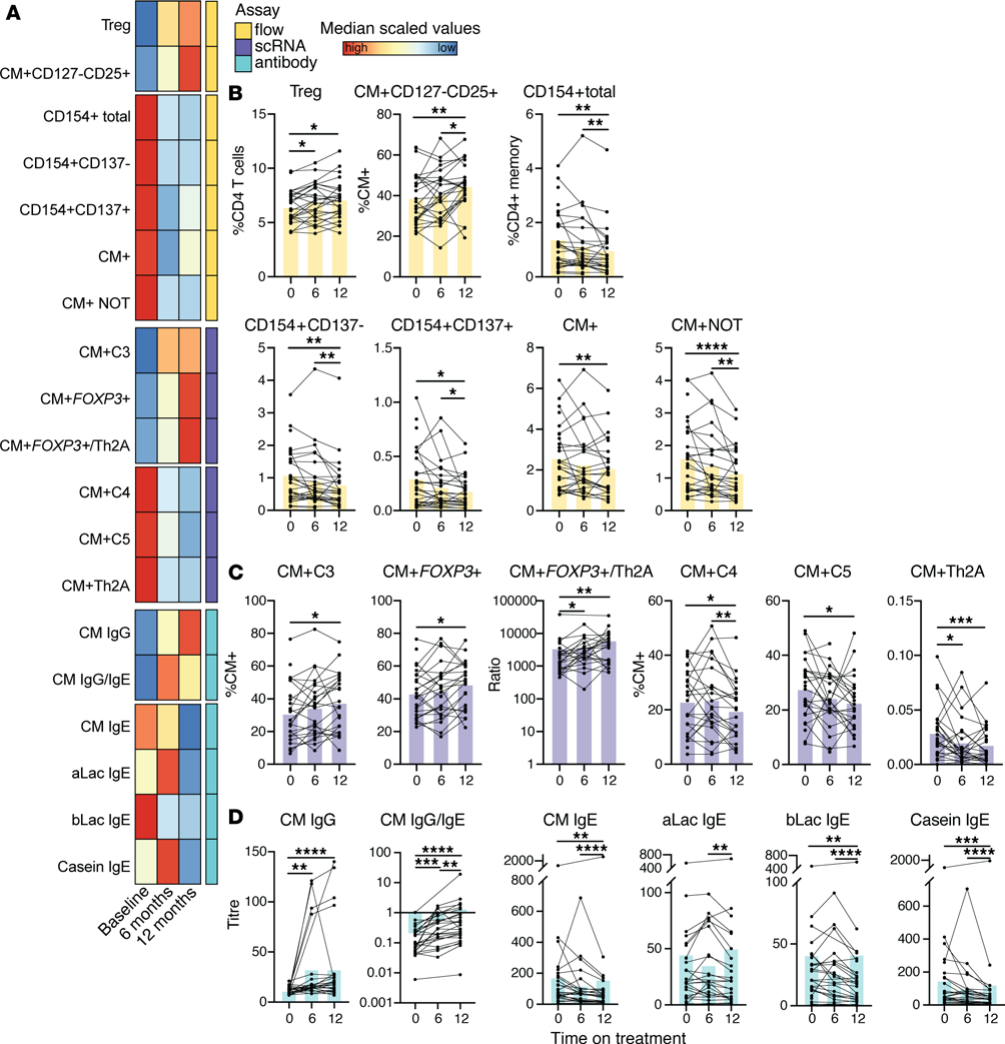
CM-specific antibody and CD4^+^ T cell populations change significantly through 12 months of BMOIT. (**A**) Heatmap showing overall changes in immune measurements over time on treatment, where color represents median value. (**B**–**D**) Bar plots with lines connecting each participant showing flow cytometry populations (**B**), scRNA-Seq populations (**C**), or antibody measurements (**D**) across time on treatment. The bars are median values. Statistical analyses were performed using paired Wilcoxon’s tests (*n* = 28). *P < 0.05; **P < 0.01; ***P < 0.001; ****P < 0.0001.

We next analyzed changes in immune measurements after 12 months of BMOIT treatment, combining both treatment arms (initial active and initial placebo) to increase statistical power. With all 28 individuals, 12 months of BMOIT treatment resulted in significant increases or decreases in all of our selected CD4^+^ T cell (flow and scRNA-Seq) populations and almost all measured antibody titers (Figure 7A). From the flow cytometry analysis, we saw significant increases in Tregs and CM^+^ CD127^–^CD25^+^ cells and decreases in CM^+^, CM^+^ NOT, CD154^+^ total, CD154^+^CD137^+^, and CD154^+^CD137^–^ populations with BMOIT (Figure 7B). For the scRNA-Seq populations, we saw significant increases in CM^+^ *FOXP3*^+^, CM^+^ C3, and the CM^+^ *FOXP3*^+^/Th2A ratio and decreases in CM^+^ Th2A, CM^+^ C4, and CM^+^ C5 (Figure 7C). For some of the populations including Tregs, the CM^+^ *FOXP3*^+^/Th2A ratio, and CM^+^ Th2A, the change in population percentage was already significant at the 6-month time point (Figure 7, B and C). Of note, there was no significant change in any T cell population from 12 months on treatment to 24 months on treatment (Supplemental Figure 6).

### CM IgE, casein IgE, β-lactoglobulin IgE, and CM IgG4 antibody levels change significantly through 12 months of BMOIT.

Antibody measurements also showed significant changes with BMOIT, with a significant increase in CM IgG4 and decreases in CM IgE, casein IgE, and β-lactoglobulin (bLac) IgE after 12 months (Figure 6D) of active treatment. The CM IgG/IgE ratio had a strong increase across OIT, with a significant increase with 6 months of BMOIT and then a further significant increase at 12 months (Figure 7D). We also found further significant decreases from 12 to 24 months on active treatment in CM and casein IgE (Supplemental Figure 6). There was no significant change in α-lactalbumin (aLac).

### No significant change in milk skin prick test.

From baseline to month 24, there was no significant change in the milk skin prick test (SPT) within or between groups. At screening, the median milk SPT wheal size was 13.5 mm (range: 7–25) for all participants. The initial BMOIT group had a decreased milk SPT wheal size from baseline (median: 14 mm, range: 7–23) to month 24 (median: 9, range: 0–19; *P* = 0.08). The placebo crossover group had an increase in milk SPT wheal size, with a change from a median of 13 mm at baseline (range: 7–25) to 17.5 mm (range: 6–24) at month 24 (*P* = 0.34). There was no significant difference in change in milk SPT wheal size over time between groups (*P* = 0.1). When grouping by time on treatment, a significant decrease in milk SPT was found after 6 months (*P* = 0.04), but not 12 months (*P* = 0.2) of active BMOIT (Supplemental Figure 7).

### Antibody and scRNA-Seq T cell population measurements best correlate with tolerated dose of BM.

**Figure 8:**
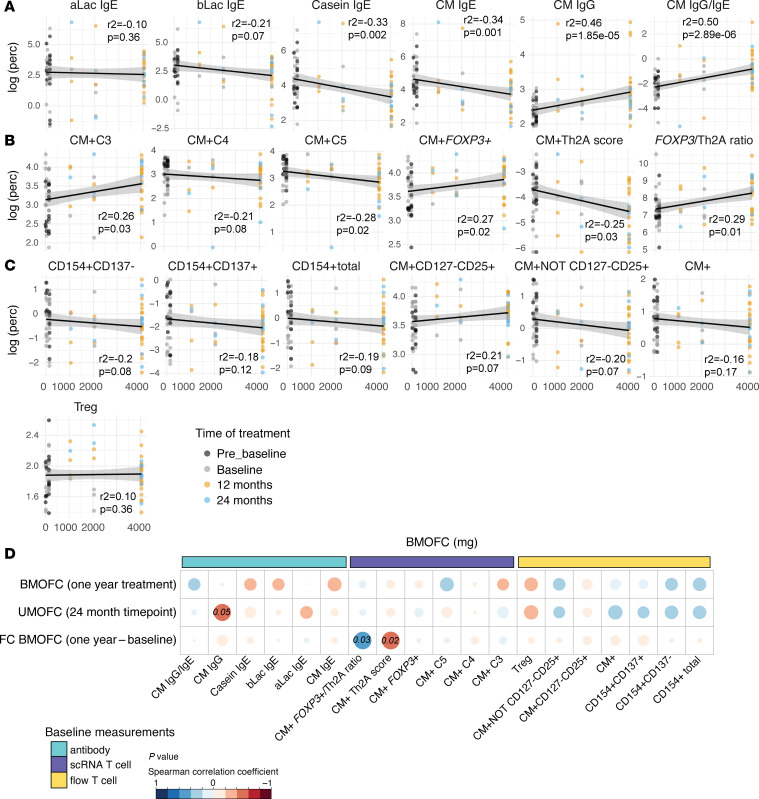
Correlations of immunologic measurements with all OFC data. (**A**–**C**) Scatter plots showing correlations of (**A**) antibody measurements, (**B**) scRNA-Seq populations, and (**C**) flow cytometry populations with baked milk oral food challenge (BMOFC) doses (mg). Color of the dot represents treatment time point. Significant correlations are noted on the plots. (**D**) Dot plot showing Spearman’s correlations of pretreatment T cell/antibody measurements with BMOFC (1 year of treatment), unheated milk oral food challenge (UMOFC) (24-month time point), or fold-change in BMOFC (1 year to baseline). Size of the dot represents magnitude of the correlation coefficient and color shows direction of correlation. Statistical analyses were performed using Spearman’s δ for those completing a BMOFC after at least 12 months on treatment (*n* = 26). Significant *P* values are noted.

We next sought to determine whether any of our immunologic measurements correlated directly with the tolerated dose of BM from OFC. Thus, we took the BMOFC tolerated doses at months 0, 12, and 24 (shown by time relative to treatment) and correlated them to their respective T cell population percentage or antibody measurement at that time point (Figure 8, A–C). The strongest correlations were observed for scRNA-Seq T cell populations and the antibody measurements. Within these, the CM IgG/IgE ratio was the most significant antibody correlation (Spearman’s *r*^2^ = 0.5, *P* = 2.89 × 10^–6^) (Figure 8A), and the CM^+^ *FOXP3*^+^/Th2A cell ratio was the most significant T cell measurement (Spearman’s *r*^2^ = 0.29, *P* = 0.01) (Figure 8B). Both measures were positively correlated with the BMOFC MTD. We additionally correlated the UMOFC MTD with the immune measurements at the challenge time point and found no statistically significant results, likely due to the single time point for the UMOFC (Supplemental Figure 8A). However, the CM^+^ C3 and CM^+^ C5 scRNA-Seq T cell populations did show trending positive and negative correlations with UMOFC, respectively (Supplemental Figure 8A). We saw no correlation of either of the OFC outcomes with baseline tolerated BM doses, age, or sex (Supplemental Figure 8B).

### Baseline proportion of CM^+^ Th2A cells negatively correlates with induction of desensitization during BMOIT.

To test whether any baseline immune measurements could predict the tolerated doses of BMOFC or UMOFC after treatment, we first performed a correlation analysis. There were no significant correlations with BMOFC, but baseline CM IgG was negatively correlated with UMOFC (*P* = 0.05) (Figure 8D). We also performed correlations with the fold-change of BMOFC (1-year treatment vs. baseline) and discovered a significant negative correlation of CM^+^ Th2A cells as well as a positive correlation with the CM^+^ *FOXP3*^+^/Th2A cell ratio (*P* = 0.02 and *P* = 0.03, respectively) (Figure 8D).

### Prediction models of OFC outcomes perform best when combining T cell and antibody measurements.

**Figure 9:**
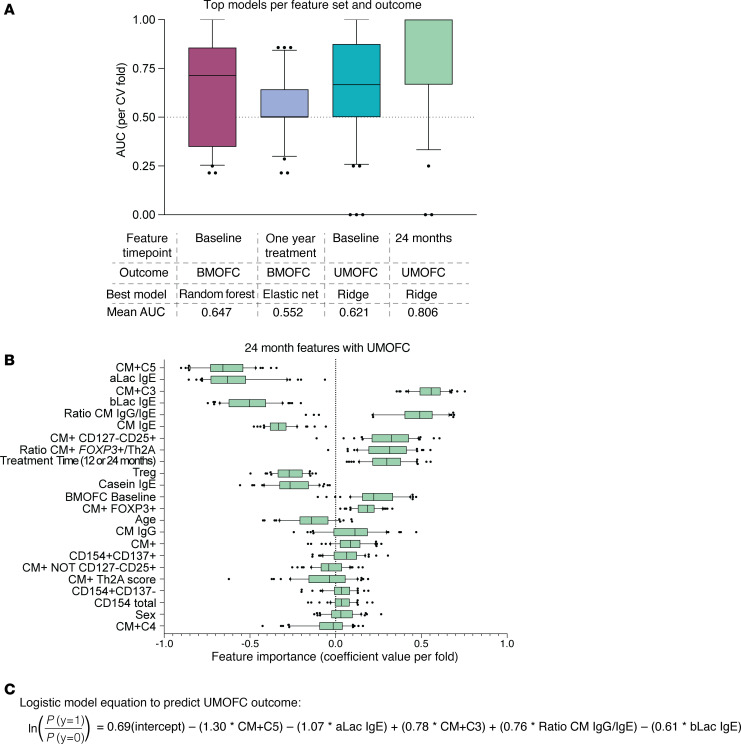
Combined classification of OFC outcome. (**A**) Box-and-whisker plots showing the area under the curve (AUC) values for each CV fold of overall best model selected (highest mean) for each set of features and outcome. Feature time point, outcome type, best model, and mean AUC across folds are indicated on the bottom. (**B**) Feature importance coefficients per fold for each feature in the best performing model from E (24-month unheated milk oral food challenge [UMOFC]). (**C**) Logistic model equation to predict UMOFC outcome. In the box-and-whisker plots, the box represents the middle 50% of data, with the bottom and top lines of the box representing the 1st and 3rd quarterlies, respectively. The line in the box represents the median. The whiskers extend to the minimum and maximum values. Outliers are indicated with dots.

Finally, we used a machine-learning approach to test the ability of the immunological measurements at baseline and challenge time points to predict desensitization using T cell, antibody, and clinical data features (Figure 9A and Supplemental Table 9). We tested this using 2 different outcome measurements based on either BM or UM OFC MTD. Cutoffs for each outcome were set to split the subjects into binary “pass”/“fail” groups for each OFC (4044 mg for BM and 2000 mg for UM). For each outcome, we tested baseline and challenge T cell and antibody measurements along with clinical features (age, sex, baseline BMOFC, and time on treatment for UMOFC only). We tested a number of different models (linear and nonlinear) with a 3-fold (BMOFC) or 5-fold (UMOFC) cross-validation approach (based on outcome imbalance) repeated 10 times. We then selected the best performing model (highest average AUC) for each outcome and set of features. We found that the highest performing model was a ridge regression that used the features at the 24-month time point to predict UMOFC outcome (mean AUC: 0.806) (Figure 9B).

We then looked at the features with the highest absolute coefficients in this top performing model for UMOFC outcome and found that a mixture of scRNA-Seq T cell (CM^+^ C5, CM^+^ C3) and antibody (aLac IgE, bLac IgE, CM^+^ IgG/IgE ratio) features were consistently more important in the model after cross-fold validation (Figure 9B). We then trained a simple logistic regression model on the full dataset (equation shown in Figure 9C), which summarizes how different factors can be combined to derive an overall score that better separates individuals with UMOFC success versus failure (AUC = 0.91, SD = 0.17) (Figure 9C). This equation could be used to predict how a participant would respond to UMOFC using inputted immunological measurements of CM^+^ C5, aLac IgE, CM^+^ C3, CM IgG/IgE ratio, and bLac IgE where the weight of each is indicated by the multiplied coefficient value.

## Discussion

This study details the treatment of severe CMA using BM rather than UM OIT. In this phase II, DBPC treatment study for CMA, BMOIT after 12 and 24 months was well tolerated and induced desensitization to BM and UM in most participants. The patients enrolled were highly sensitive (reacted to <444 mg of BM at baseline) and unlikely to tolerate BM introduction in a clinic setting given their low reaction threshold. After the initial year of treatment, we found that 11 of 14 (79%) in the BMOIT group compared with 0 of 14 in the placebo group could tolerate 4044 mg of BM protein during the 12-month BM DBPCFC (25). During the second year of the study, all participants were on active BMOIT. Nineteen of 30 (63%) reached the primary endpoint of tolerating 4044 mg of BM protein at the 24-month BM DBPCFC (79% in the PP analysis), and 13 of 30 (43%) tolerated 2000 mg or more of UM (59% in the PP analysis). In the PP analysis, those on 24 months of treatment tolerated more UM compared with those receiving 12 months of active BMOIT, suggesting that there may be increased efficacy in longer duration of treatment.

There have been limited studies of BM OIT in BM-reactive children, with our trial being the only DBPC randomized control trial of BM OIT in BM reactive children to date to our knowledge (19, 25, 29, 30). A study by Goldberg et al. of 15 BM-reactive patients found that only 3 of 14 (21%) tolerated the goal dose of 1.3 g of BM (19). In a retrospective analysis, Zhang et al. found that 12 of 18 (67%) of BM-reactive children reached maintenance dosing (target dose of 667 to 1330 mg) (29). Our study found that 26 of 30 (87%) reached the maintenance dose of 2 g BM. The increased success in our study is likely due to the lower starting dose and more gradual dose escalation.

This study found that BM exposure can lead to nonreactivity to less-cooked forms of milk. Gruzelle et al. found that 27 of 64 children (42%) tolerated 254 mL of liquid milk (8.6 g milk protein) at an average duration of 521 days of BM OIT (30). The higher percentage achieving desensitization to 8 g of UM compared with our study (4 of 30 [13%]) is likely because only 21 of 64 (33%) reacted during the low-dose BM OFC prior to starting BM OIT. In addition, the mean age was 4.8 years and natural resolution may have been more likely in this younger group. Amat et al. randomized raw milk–allergic patients to raw milk OIT or BM OIT and found no difference in gain of tolerance or AEs between OIT arms (31).

Regarding safety, our BMOIT protocol appears to be well tolerated, with greater than 98% of AEs being mild. During study year 1, symptoms were reported with 42% of active BMOIT doses. During open-label treatment in study year 2, dose-related symptoms were reported with 12% of BMOIT doses. Symptoms were reported in 8 of 13 (61.5%) and 14 of 14 (100%) of those in the initial active and placebo crossover groups, respectively. Dose-related symptoms decreased in year 2 compared with year 1 for the initial active group, supporting the idea that safety likely increases over time, although reactions can still occur. Throughout the entire 24-month period, 6 participants discontinued, but only 1 participant discontinued solely due to symptoms with OIT and 2 stopped primarily due to starting college, but also because of symptoms with dosing. Direct safety comparison to other studies or unheated OIT is difficult because of the distinctive nature of this patient population and study protocol. However, our lower rate of withdrawals and rarity of moderate or severe reactions indicates that BMOIT may be more tolerable than traditional OIT (1, 32–36).

Using serological measures of desensitization, we found that 12 months of active BMOIT was associated with significant decreases in CM IgE, casein IgE, and bLac IgE, with further significant increases in CM IgE and casein IgE from 12 to 24 months. Consistent with previous studies and our findings from year 1, we found that both groups had an increase in CM IgG4 and the CM IgG4/IgE ratio after 12 months of active treatment (1, 25, 35). The CM IgG4/IgE ratio was also significantly correlated with BM OFC MTD.

In addition to the serologic studies, we also monitored antigen-specific T cell responses using our previously published method (28). CM^+^ CD4^+^ memory T cells were isolated and profiled longitudinally through BMOIT using flow cytometry and scRNA-Seq. To produce findings from such high-dimensional datasets that we have confidence in and believe would transfer to subsequent trials, we used a hypothesis-driven analytical approach. Specifically, we preselected T cell populations that we had previously shown to be associated with allergic status in cross-sectional studies, that had been previously associated with tolerance, and/or were defined by antigen specificity. We hypothesized that changes in these populations would be associated with the development of desensitization through OIT.

We aimed to answer 3 major questions through this analysis with increasing levels of stringency and relevance. The first was whether we could see changes in T cell populations that predict BMOIT or placebo group membership for individuals after 1 year. The best predictor in our dataset was the CM^+^ *FOXP3*^+^/Th2A cell ratio from our scRNA-Seq analysis, which outperformed flow cytometry as well as CM IgG or IgE measurements. In fact, the scRNA-Seq measurements in general outperformed the others, suggesting that changes in gene expression of CM^+^ cells are a more robust indicator for BMOIT-induced desensitization. Similar findings have been published recently where scRNA-Seq analysis showed significant changes in antigen-specific T cell populations through OIT for peanut allergy (37).

The second question addressed which of the immunological measures changed over the course of 1 year of BMOIT. We found significant redistribution of T cell subsets with OIT where both flow cytometry and scRNA-Seq subsets showed increases in Treg and CM^+^ *FOXP3*^+^ populations and associated decreases in CM^+^ Th1/17/2 subsets and overall CM^+^ cells. Our previous work had shown that increased CM^+^ *FOXP3*^+^ and Th2A populations distinguish CM-allergic versus non–CM-allergic individuals. We found here that the CM^+^ Th2A cells, defined by pathogenic Th2 and Tfh genes, decreased through BMOIT. Whether these cells are becoming anergic or being depleted is unclear and is being investigated. While similar findings on pathogenic Th2A cells have been published for other allergies (37–42), the role of antigen-specific *FOXP3*^+^ populations is less clear (22). We show here that CM^+^ *FOXP3*^+^ cells increase significantly with 1 year of BMOIT treatment. This is mirrored in the flow cytometry data where we show increases in both antigen-specific (CM^+^ CD127^–^CD25^+^) and non–antigen-specific Tregs. This suggests that these cells may have a suppressive role in BMOIT-induced desensitization. Future studies are needed to define the functional role of these populations. Of note, we did not find any significant differences between 1 year and 2 years on treatment in terms of T cell populations. Longer-term studies will need to be performed to assess the longevity of tolerance, especially if OIT is stopped.

Finally, the third question asked was whether we were able to predict desensitization based on immunological measurements. To determine the relationship between immunological measurements and clinical determinants of desensitization, we performed correlations of our T cell and antibody measurements with BM and UM OFC dose values taken at each food challenge time point (0, 12, and 24 months for BM and 24 months for UM). We identified T cell populations (scRNA-Seq) and antibody measurements had more significant correlations, with the most significant being the CM^+^ *FOXP3*^+^/Th2A ratio for T cells and the CM IgG/IgE ratio for antibodies. This confirms the already known role of IgG/IgE measurements in diagnostics as well as points again to the significance of CM^+^ *FOXP3*^+^ and Th2A cells and their potential uses in defining desensitization.

We also used machine learning to predict desensitization outcomes. This analysis revealed that a model combining challenge time point features from multiple measurement types (antibody, T cell) could predict UMOFC outcomes with high accuracy in our cohort. This would be a major benefit, as it would reduce the need for food challenges, which are resource intensive and pose risks. The features with the highest importance across folds included CM^+^ C5 (T cell scRNA-Seq), aLac IgE, CM^+^ C3 (T cell scRNA-Seq), and bLac IgE. From the final model equation, decreases in CM^+^ C5, aLac IgE, and bLac IgE and accompanying increases in CM^+^ C3 would yield higher desensitization to UM. The scRNA-Seq population CM^+^ C5 was *FOXP3*^–^ and showed high expression of activated T cell– and Th1/Th17–skewing markers (*CCL20*, *CD69*, *TNF*, *MIR155HG*, *CD40LG*, and *NFKB1*). The CM^+^ C3 cluster was *FOXP3*^+^ and showed high expression of activated Treg and MHC II markers (*HPGD*, *TIGIT*, *HLA-DRB1*, and *HLA-DPA1*). So, decreased antigen-specific Th1/17 cells and aLac and bLac IgE accompanied by increased antigen-specific, activated MHC II^hi^ Tregs is predictive of higher desensitization to UM. We acknowledge that this predictive model is preliminary and should be validated with an external cohort. We also acknowledge the challenges of using scRNA-Seq in a clinical setting and plan to follow these findings up and attempt to find surface markers or different measurement methods for these rare T cell subsets.

The study had some limitations. The 12-month and 24-month BM DBPCFC had a maximum cumulative dose of 4044 mg and since most tolerated that amount, we are unable to explore variables related to tolerated dose or comparison to UM amount. In addition, we were unable to directly compare safety and efficacy to UM OIT. Finally, this study had a small sample size, which was appropriate for this proof-of-concept design, but will need to be replicated in larger studies for more generalizability.

In summary, this study showed that most patients with severe CMA could be desensitized to BM and UM and that overall, our protocol appears to be safe and well tolerated. Mechanistically, desensitization to CM seems to be at least in part mediated by increases in CM IgG4 and CM^+^ *FOXP3*^+^ T cell populations and corresponding decreases in CM IgE and CM^+^ pathogenic Th2 populations, which is an interesting finding in the CMA field.

## Methods

### Sex as a biological variable.

This study involved both male and female participants. We did evaluate sex as a biological variable for clinical and laboratory outcomes.

### Trial design.

As previously described, this clinical trial included a screening phase (including a DBPCFC) to up to 444 mg of BM protein), a blinded phase of BM OIT versus placebo that lasted approximately 12 months (including an IDE day), buildup, and maintenance period, and then a 12-month DBPCFC to up to 4044 mg of BM protein), unblinding, and then an open-label BM OIT phase that lasted an additional 12 months (Figure 2 and Supplemental Tables 7 and 8) (25). After the study was unblinded, the initial placebo group crossed over to active therapy utilizing the same protocol followed in year 1 (IDE day, buildup to 2000 mg of BM protein, and then maintenance phase). The initial BM group continued on 2000 mg of BMOIT for an additional 12 months. At month 24, participants underwent a DBPCFC to up to 4044 mg of BM protein. If they tolerated 2000 mg or more, they then underwent a UM DBPCFC to up to 8000 mg of UM. All participants were then given recommendations for home milk introduction with a plan to follow-up for an additional 24 months. The full protocol can be obtained by contacting the corresponding author.

### Participant selection, randomization, and blinding.

All participants were recruited from the Johns Hopkins (JH) Pediatric Allergy clinic (Baltimore, Maryland). Participants were males and females age 3 to 18 years with a history of symptomatic reactivity to CM; milk SPT wheal diameter at least 3 mm greater than the negative control; and CM IgE greater than 5 kU/L. Participants were required to have dose-limiting symptoms to 444 mg or less of BM protein during the screening DBPCFC. Qualifying participants were randomized 1:1 to BMOIT or placebo OIT for the initial 12 months of treatment. The study team and participants were blinded to treatment until after month 12 of DBPCFC, at which time unblinding occurred. Full inclusion and exclusion criteria and additional details about randomization, blinding, and sample size have been previously published (25) and are detailed in the supplemental material.

### Study product and dosing protocol.

The milk and placebo powders were supplied by the University of North Carolina, provided as individually packaged doses for dispensing and bulk product for use by JH nutritionists. Measured doses were provided in individual cups for all doses consumed at home. Participants were given instructions on how to prepare the OIT dose at home, including preparing a cupcake or muffin batter, mixing the premeasured OIT powder in a regular size muffin tin with batter, and baking for 30 minutes at 350 degrees. All doses given during a food challenge or in the JH pediatric clinical research unit (PCRU) were prepared by the JH Research Nutrition Team according to prespecified cake recipes. Additional information related to the study product can be found in the supplemental material.

The OIT protocol began with a single-day IDE day in the PCRU (Supplemental Table 7). Participants then began daily OIT home dosing with doses of 3 to 25 mg of BM protein or placebo. They returned to clinic every 10–21 days for further dose increases (buildup period) up to the target maintenance dose of 2000 mg and then every 2 months during year 1.

During the unblinded second year of the trial, all participants received BMOIT. All those in the initial BMOIT group who completed the blinded phase reached the target maintenance dose of 2000 mg prior to month 12 of DBPCFC. These participants continued on 2000 mg of BM protein daily for an additional 12 months with visits every 3 months. Those initially on placebo underwent a single-day IDE, during which increasing OIT doses were administered as tolerated, starting with 0.1 mg up to a maximum of 25 mg of BM milk protein (cumulative of 44 mg) (Supplemental Table 7). They then began daily BMOIT dosing and returned every 10–21 days for further dose increases up to the target maintenance of 2000 mg (minimum dose requirement of 750 mg) (Supplemental Table 7) and then every 2 months during maintenance. All participants were instructed to take the same dose at home every day between visits and keep a daily dosing diary. Maintenance dosing was continued for at least 8 weeks prior to the month 24 DBPCFC.

The COVID-19 pandemic impacted the year 2 study protocol for 18 participants, as described in the supplemental material.

### Study outcomes.

The primary outcome for the second year of the study was the tolerance of 4044 mg of cumulative BM protein without dose-limiting symptoms in a DBPCFC after 12 and 24 months of treatment. Secondary endpoints included tolerance of up to 8 g of UM at month 24, incidence of AEs, change in MTD of BM from baseline to month 24, differences in clinical response based on duration of treatment, exploration of biomarkers, and mechanistic correlates of desensitization.

### DBPCFCs.

The baseline BM food challenge was performed as a DBPCFC, with the active portion consisting of cake with a cumulative 444 mg of BM protein. The month 12 and 24 BM DBPCFCs had a cumulative dose of 4044 mg (Supplemental Table 8). The month 24 UM challenge had a maximum cumulative dose of 8030 mg. Additional details about the DBPCFCs has been previously published (25) and can be found in Supplemental Table 8.

### Safety assessments.

Participants recorded dosing symptoms and any medications used on a daily home diary log. Dosing logs were reviewed by study personnel at each visit. AEs, serious AEs, and accidental exposures to milk were reported throughout the study and graded using the CoFAR grading system for allergic reactions (27).

### Immune studies.

Blood for mechanistic studies was collected at baseline, start of maintenance (year 1 and year 2 for placebo-crossover group), and day 1 of the month 12 and month 24 DBPCFCs. IgE against CM, aLac, bLac, casein, and IgG4 against CM were measured by ImmunoCap (Thermo Fisher Scientific) at the JH Dermatology, Allergy, and Clinical Immunology reference laboratory. SPT was performed using CM extract from Greer Laboratories. SPT score was calculated as the milk wheal size minus the saline control wheal size.

### PBMC isolation.

PBMCs were isolated by density gradient centrifugation (Ficoll-Hypaque, Amersham Biosciences) from the obtained blood, cryopreserved at a concentration ranging between 1 × 10^7^ and 2 × 10^7^ cells/mL in FBS (GeminiBio) plus 10% DMSO (Sigma-Aldrich), and shipped to the La Jolla Institute for Immunology as previously described (43).

### Activation and sorting of memory CD4^+^ T cells for scRNA-Seq.

In order to identify and isolate CM^+^ T cells based on M111 peptide pool stimulation (28), FACS isolation of CD154^+^ and/or CD137^+^ memory CD4^+^ T cells from PBMCs was conducted as previously described (28, 44, 45). Briefly, PBMC vials of 20 study participants were thawed and plated at a concentration of 20 million cells/mL in 96-well plates and incubated overnight at 37°C with 5% CO_2_ in HR5 media (RPMI 1640 [Omega Scientific] supplemented with 5% human AB serum [Gemini Bio-Products], 1% penicillin/streptomycin [Gemini Bio-Products], and 1% Glutamax [Gibco by Life Technologies]). Blocking anti-CD40 antibody (1 μg/mL; Miltenyi Biotec, 130-108-041) and anti-CD28 costimulatory antibody (eBioscience, 14-0289-82) were added and cells were stimulated with DMSO (1:250, negative control), phytohemagglutinin (PHA, 1 μg/mL; positive control), and M111 peptide pool (2 μg/mL) for 6 hours. The cells were then harvested and washed in PBS (pH 7.4, Gibco) plus 10% FBS. The cells were then resuspended in 100 μL of a cocktail containing viability dye (eFluor 506, eBioscience, 65-0886-14), FcR blocking TruStain FX (BioLegend, 422302), anti-CD4 (APCef780, clone RPA-T4, Thermo Fisher Scientific, 47-0049-42), anti-CD3 (Alexa Fluor 700, clone UCHT1, Thermo Fisher Scientific, 56-0038-42), anti-CD8/-CD14/-CD19/-CD56 (V500, clones RPA-T8, M5E2, H1B19, NCAM16.2, all from BD Biosciences, 560774, 561391, 561121, 563041), anti-CD45RA (eFluor 450, clone HI1000, BD Biosciences, 48-0458-42), anti-CCR7 (FITC, clone G043H7, BioLegend, 353216), anti-CD154 (PE, 24-31, BioLegend, 310806), anti-CD137 (APC, clone 4B4-1, Thermo Fisher Scientific, 309810), anti-CD25 (BV605, clone 2A3, BD Biosciences, 562660), and anti-CD127 (PE-Cy7, clone eBioRDR5, Thermo Fisher Scientific, 25-1278-42). To allow sample multiplexing, 2 μL of BioLegend Total-seq C DNA-oligo-conjugated antibodies (TotalSeq anti-human Hashtags 1–10, 12–20, and 24) were added. After 30 minutes of surface staining at 4°C, cells were washed 3 times in FACS buffer and resuspended at a concentration of 20 ×10^6^ cells/mL in FACS buffer and kept in ice until sorting. Memory CD4^+^ T cells expressing CD154 and/or CD137 (CM^+^) were FACS isolated using 2 FACSAria II machines concurrently (BD Biosciences). We also sorted CD154^–^CD137^–^ cells (CM^–^) to be able to compare T cell heterogeneity of CM^+^ CD4^+^ cells with the overall CD4^+^ population. The sorting strategy is summarized in Supplemental Figure 3. All flow cytometry data were analyzed using OMIQ software (https://www.omiq.ai/).

### scRNA-Seq library preparation and sequencing.

For scRNA-Seq assays, we used the 10× Genomics platform. For each patient sample, approximately 2,500 CM^+^ memory T cells and approximately 3,500 CM^–^ cells were collected in low-retention and sterile ice-cold 1.5 mL tubes containing 500 μL of PBS/FBS (1:1 volume) completed with RNase inhibitor (1:100). Each experiment of 20 samples (all time points for a given individual were in the same run) generated 2 tubes where each combined the CM^–^ fraction of 10 samples with the CM^+^ fraction of 10 different samples. Approximately 60,000 sorted cells per tube were processed and loaded on the 10× Chromium Controller (10× Genomics). We used a 5′ mRNA capture chemistry (5′ 10× v2 chemistry) and performed cDNA amplification and library preparation for gene expression and feature barcoded surface antibodies. Final libraries were quality checked for fragment size by capillary electrophoresis (fragment analyzer), and quantity by fluorescence assay (Picogreen) before pooling and sequencing on a NovaSeq 6000 (200 cycle S4 kit v1.5; Illumina) to a depth of greater than 25,000 reads/cell for gene expression, greater than 5,000 reads/cell for hashtag oligonucleotide libraries.

### scRNA-Seq analysis — hashtag demultiplexing, QC filtering, clustering, module scores.

Sequencing reads were aligned to the GRCh38 human reference genome using the “multi” pipeline in CellRanger (v5.0; 10× Genomics). Downstream analysis was performed using the package Seurat (v5; https://satijalab.org/seurat/index.html) in R (v4.2.2). To overcome donor-specific gene expression of individual TCR genes, TCRA/B/D/G genes were pulled from the gene expression matrix and counts were aggregated into a single gene feature for each. Demultiplexing was performed using MULTIseqDEMUX on merged lanes from each experiment day to overcome hashtag imbalances (46). Cells called as doublets or negatives and those with high mitochondrial content (>5%) and/or low feature number (<500) and/or high RNA counts (>20,000) were removed for downstream analysis. After filtering, we retrieved 150,470 cells (Supplemental Figure 3), which were then normalized using SCTransform with parameters to regress out mitochondrial percentage, to remove genes with low expression from normalization using “v2” regularization, and using 2000 variable features. Integration of the datasets was performed using the RPCA integration function in Seurat to remove batch effects between runs. Principal component analysis was performed using the RunPCA function with the top 30 PCs. RunUMAP and FindNeighbors were performed with 30 dimensions and a k.param of 30. Finally, FindClusters was applied and identified 23 clusters (Supplemental Figure 4A). The CM^+^ sorted cells clustered closely together in clusters 3, 4, 5, 10, 18 and 22, which were made up of more than 80% cells from the CM^+^ sorted fraction as compared with CM^–^ (Supplemental Figure 4, A and B). Cluster-specific markers were obtained using the FindAllMarkers function with default parameters. All visualization plots were produced in Seurat or ggplot2. Module scores were added for selected gene lists using the AddModuleScore function in Seurat.

### Machine learning.

We aimed to identify immune measurements that would predict DBPCFC outcomes. Cutoffs were set for each outcome, categorizing each participant into a pass or fail group depending on MTD (>4000 mg for BMOFC, >2000 mg for UMOFC). We tested both linear (logistic regression, elastic net, ridge regression) and nonlinear (decision tree, random forest) machine-learning methods with different parameters in the scikit-learn package (v1.2.2; https://scikit-learn.org/stable/index.html). For each set of features (baseline or challenge) and outcome (BM or UM) the best performing model and parameters was selected. Prior to training, missing values were replaced with median values and feature normalization (via *z*-score normalization) was applied to ensure compatibility across modalities. We used a 3-fold (BMOFC) or 5-fold (UMOFC) cross-validation approach repeated 10 times. Number of folds was chosen based on numbers of participants in pass/fail categories. Performance of each model fold was assessed using AUC and the mean AUC was used to determine the best performing model for each set of features and outcome. Model details can be found in Supplemental Table 9.

### Statistics.

All assessments were done in the ITT population (*n* = 30) unless stated otherwise. Tolerated dose was imputed as 0 mg for participants who did not complete the month 24 BM or UM DBPCFCs. The PP sample for desensitization was defined as all-intention ITT participants who adhered to maintenance dosing per protocol and had an evaluable DBPCFC at month 24. The primary outcome, desensitization to 4044 mg BM, was evaluated using Fisher’s exact test. We also calculated the proportion of participants who tolerated a cumulative of 2044 mg and 8030 mg of UM at month 24. Those who did not complete the month 24 DBPCFC (*n* = 6) were considered to not have a change in MTD. The within-group difference in MTD from baseline to month 24 and between group change in MTD were compared using Wilcoxon’s signed-rank and Mann-Whitney *U* tests, respectively.

Participant AE occurrence and dose with symptom rates were summarized overall and by trial phase (buildup and maintenance). Dosing-related symptom rates were compared between groups and are reported as incidence rate ratios with corresponding 95% CIs based on exact procedures.

Laboratory values at baseline through month 24 were summarized and the within- and between-group changes were compared using the previously described methods. For analyses using time-1 treatment, end of year 1 values were considered time 0 for the placebo crossover group since these were the values just prior to starting BMOIT. Two-way comparisons were performed by either Mann-Whitney *U* test (unpaired) or Wilcoxon’s signed-rank test (paired). The Benjamini, Krieger, and Yekutieli 2-stage test for multiple comparisons was used when necessary. Correlations were performed using Spearman’s δ, a nonparametric analysis. *P* or FDR values of less than 0.05 were considered statistically significant. All analyses were performed with STATA 18 (StataCorp) or GraphPad Prism (v9).

### Study approval.

This study was approved by the JH (Baltimore, Maryland) and La Jolla Institute of Immunology (La Jolla, California) Institutional Review Boards. The study was registered on ClinicalTrails.gov (NCT03462030) and was conducted under a US Food and Drug Administration investigational new drug application. Written informed consent was obtained from parents/guardians with assent from those older than 6 years, and directly from participants 18 years of age or older.

### Data availability.

Sequencing data are accessible online through the NCBI Gene Expression Omnibus (accession number GSE280063, https://www.ncbi.nlm.nih.gov/geo). Other data are available in the main text or the supplemental material. Raw data supporting the conclusions of the study can be found in the Supporting Data Values file. Additional data can be obtained by contacting the corresponding author and will be provided after deidentification, in compliance with applicable privacy laws, data protection, and requirements for consent and anonymization.

## Author contributions

All authors contributed to the study design or the data acquisition, analysis, and interpretation. JAD and RAW conducted the clinical trial, including recruitment and study visits. SAL, AS, AF, SM, and BP contributed to the procurement of clinical samples and conducting of laboratory experiments. All authors drafted or critically revised the work and read and approved the final manuscript. First authorship order was assigned based on degree of involvement in the clinical trial.

## Supplementary Material

Supplemental data

ICMJE disclosure forms

Supporting data values

## Figures and Tables

**Table 1:**
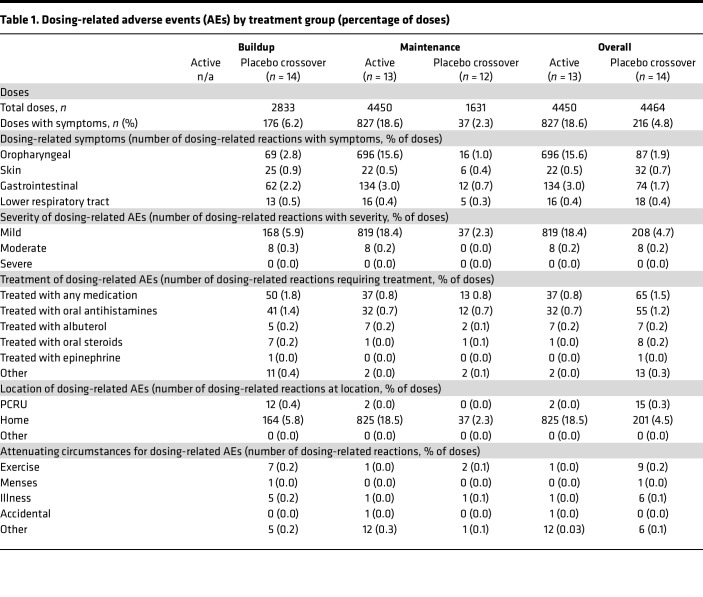
Dosing-related adverse events (AEs) by treatment group (percentage of doses)

## Version 1 01/09/2025

Electronic publication
