# Supplementary material for: Clinical and immunological outcomes after randomized trial of baked milk oral immunotherapy for milk allergy
Source: JCI Insight. 2025 Jan 9;10(1):e184301. doi: 10.1172/jci.insight.184301 (PMC11721308; doi:10.1172/jci.insight.184301)
Supplement: Supplemental data [file jciinsight-10-184301-s215.pdf]

Supplemental Data

I. Supplemental Tables

A. Table S1. Baseline demographic and allergy characteristics of participants

B. Table S2. Percentage of participants successfully tolerating predefined cumulative dose during the month-24 oral food challenges

C. Table S3. Summary of all adverse events, according to Trial Phase (number of participants with event)

D. Table S4. Difference in dosing-related reactions in year 2 vs year 1 of baked milk OIT for the initial active group (percentage of doses)

E. Table S5. Epinephrine use during the second year of the trial

F. Table S6. Detailed description of T cell populations selected for analysis

G. Table S7. Baked milk oral immunotherapy dosing schedule

H. Table S8. Dosing Schedule for Double-Blind, Placebo-Controlled Food Challenges

I. Table S9. Detailed model parameters

II. Supplemental Figures

A. Figure S1. Maximum cumulative tolerated dose of baked milk by time on treatment

B. Figure S2. Percent tolerating dose of unheated milk during month-24 DBPCFC

C. Figure S3. Gating strategy for sort and flow cytometry analysis

D. Figure S4. scRNA-Seq supplemental

E. Figure S5. T cell and antibody measurements by treatment group across all time point

F. Figure S6. Population changes from 12 to 24 months on treatment

G. Figure S7. Skin prick test

H. Figure S8. Correlation analysis supplemental

III. Supplemental Appendices

1. Full inclusion/exclusion Criteria

2. Study product details

3. Double-blind, placebo-controlled food challenge details

4. Sample Size, Randomization, and Blinding

5. Impact of COVID-19 pandemic

Supplemental Tables

Table S1. Baseline demographic and allergy characteristics of participants

	**Milk OIT (n=15)**	**Placebo group (n=15)**	**All randomized participants (n=30)**
**Sex** (n (%))*			
Male	7 (47%)	9 (60%)	16 (53%)
Females	8 (53%)	6 (40%)	14 (47%)
**Median age**, years (range)	13 (4-18)	7 (3-14)	11 (3-18)
**Race** (n (%))*			
White	12 (80%)	9 (60%)	21 (70%)
Black	2 (13%)	2 (13%)	4 (13%)
Asian	1 (7%)	2 (13%)	3 (10%)
Native Hawaiian or Other Pacific Islander	0	0	0
American Indian or Native Alaskan	0	0	0
Multiple races	0	2 (13%)	2 (7%)
**Ethnicity***			
Hispanic or Latino	0	1 (7%)	1 (3%)
Not Hispanic or Latino	15 (100%)	14 (93%)	29 (97%)
**Milk History**			
Age at milk allergy diagnosis (months) (median, range)	8mo (4-14mo)	9mo (4-24mo)	8.5mo (4-24mo)
Prior Baked Milk Exposure	12 (80%)	10 (67%)	22 (73%)
History of milk reaction			
Unheated only	3 (20%)	5 (33%)	8 (27%)
Baked only	3 (20%)	0	3 (10%)
Both	9 (60%)	10 (67%)	19 (63%)
**Other current atopic history** (n (%))			
Asthma	11 (73%)	9 (60%)	20 (67%)
Atopic dermatitis	8 (53%)	8 (53%)	16 (53%)
Allergic rhinitis	10 (67%)	11 (73%)	21 (70%)
Non-milk food allergies	12 (80%)	14 (93%)	26 (87%)
**Laboratory and Skin Testing (**median, range)			
Cow milk IgE (kU/L)	114 (7.2-1625)	86.8 (21.6-772)	87.65 (7.2-1625)
Alpha lactalbumin IgE (kU/L)	20.4 (0.6-556)	19.7 (0.2-291)	20 (0.2-556)
Beta lactoglobulin igE (kU/L)	28.2 (0.4-487)	18.8 (4.1-79.3)	24.5 (0.4-487)
Casein IgE (kU/L)	75.8 (5.9-1326)	52.8 (15.4-652)	64.8 (5.9-1326)
Milk IgG4 (mg/L)	8.6 (6.9-12.5)	9.4 (7.4-19)	9.2 (6.9-19)
Milk skin prick test (mm)	14 (7-23)	13 (7-25)	13.5 (7-25)
**Screening OFC- Maximum Cumulative Tolerated Dose**[+] of Baked Milk Protein			
4 mg	1 (6%)	2 (13%)	3 (10%)
14 mg	4 (27%)	1 (6%)	5 (17%)
44 mg	4 (27%)	2 (13%)	6 (20%)
144 mg	6 (40%)	10 (67%)	16 (53%)

*The participant made the classification for sex, race, and ethnicity. Options were defined by the investigator.

Table S2. Percentage of participants successfully tolerating predefined cumulative dose during the month-24 oral food challenges

	N	Initial Active	Placebo cross-over	Difference (95% CI)	p-value⁺
**Intent-to-Treat***					
**Baked Milk**	30				
4044 mg		9/15 (60%)	10/15 (67%)	7% (-0.3,0.4)	1
**Unheated Milk**	30				
2000 mg		8/15 (53%)	5/15 (33%)	20% (-0.5, 0.2)	0.46
8030 mg		4/15 (27%)	0/15 (0%)	27% (-0.49,-0.04)	0.1
**Per Protocol**					
**Baked Milk**	24				
4044 mg		9/12 (75%)	10/12 (83%)	8.3% (-0.2,0.4)	1
**Unheated Milk**	22				
2000 mg		8/11 (73%)	5/11 (45%)	27% (-0.67,0.12)	0.39
8030 mg		4/11 (36%)	0/11 (0%)	36% (-0.65,-0.08)	0.09

⁺p-value- Fisher-Exact

* Tolerated dose was imputed as 0 mg for participants who did not complete the month-24 BM or unheated milk DBPCFCs.

Table S3. Summary of all adverse events, according to trial phase (number of participants with event)

	Initial Dose Escalation		Build up		Maintenance		Overall	
	active **N/A**	Placebo Cross-over (n=14)	active **N/A**	Placebo Cross-over (n=14)	active (n=13)	Placebo Cross-over (n=12)	active (n=13)	Placebo Cross-over (n=14)
**Participants with Adverse Event (n,%)**								
≥ 1 Adverse Event		4 (28.6)		14 (100)	9 (69.2)	11 (91.7)	9 (69.2)	14 (100)
≥ 1 Non-dosing related AE		2 (14.3)		13 (92.9)	6 (46.2)	3 (25.0)	6 (46.2)	14 (100)
≥ 1 Dosing-related AE		3 (21.4)		14 (100)	8 (61.5)	9 (75.0)	8 (61.5)	14 (100)
**Symptoms (n,%)**								
Oropharyngeal symptoms		2 (14.3)		8 (57.1)	4 (30.8)	6 (50.0)	4 (30.8)	9 (64.3)
Symptoms other than oropharyngeal								
---Skin		2 (14.3)		8 (57.1)	5 (38.5)	4 (33.3)	5 (38.5)	9 (64.3)
---GI		0 (0)		13 (92.9)	8 (61.5)	3 (25.0)	8 (61.5)	13 (92.9)
---Respiratory		0 (0)		10 (71.4)	6 (46.2)	3 (25.0)	6 (46.2)	10 (71.4)
**Severity of adverse event (n,%)**								
---Mild		4 (28.6)		14 (100)	9 (69.2)	11 (91.7)	9 (69.2)	14 (100)
---Moderate		0 (0)		4 (28.6)	3 (23.1)	1 (8.3)	3 (23.1)	4 (28.6)
---Severe		0 (0)		0 (0)	0 (0)	0 (0)	0 (0)	0 (0)
**Treatments (n,%)**								
---Treated with any medications		3 (21.4)		14 (100)	8 (61.5)	6 (50.0)	8 (61.5)	14 (100)
---Treated with oral antihistamines		3 (21.4)		11 (78.6)	6 (46.2)	5 (41.7)	6 (46.2)	12 (85.7)
---Treated with albuterol		0 (0)		6 (42.9)	2 (15.4)	2 (16.7)	2 (15.4)	7 (50.0)
---Treated with oral steroids		0 (0)		3 (21.4)	1 (7.7)	1 (8.3)	1 (7.7)	3 (21.4)
---Treated with epinephrine		0 (0)		2 (14.3)	0 (0)	1 (8.3)	0 (0)	3 (21.4)
Other		1 (7.1)		11 (78.6)	3 (23.1)	3 (25.0)	3 (23.1)	11 (78.6)
**Location (n,%)**								
Clinical Research Unit		3 (21.4)		6 (42.9)	1 (7.7)	0 (0)	1 (7.7)	7 (50.0)
Home		2 (14.3)		14 (100)	9 (69.2)	11 (91.7)	9 (69.2)	14 (100)
Other		0 (0)		0 (0)	1 (7.7)	1 (8.3)	1 (7.7)	1 (7.1)
**Attenuating Circumstances (n,%)**								
Exercise		0 (0)		4 (28.6)	1 (7.7)	2 (16.7)	1 (7.7)	5 (35.7)
Menses		0 (0)		1 (7.1)	0 (0)	0 (0)	0 (0)	1 (7.1)
Illness		0 (0)		5 (35.7)	4 (30.8)	3 (25.0)	4 (30.8)	7 (50.0)
Accidental Ingestion		0 (0)		2 (14.3)	1 (7.7)	0 (0)	1 (7.7)	2 (14.3)
Other		0 (0)		4 (28.6)	2 (15.4)	1 (8.3)	2 (15.4)	5 (35.7)

Abbreviations: AE- adverse event

Table S4. Difference in dosing-related reactions in year 2 vs year 1 of baked milk OIT for the initial active group (percentage of doses)

	**Year 1**	**Year 2**	**IRR (95% CI)**
**Doses**			
Total Doses (n)	5277	4450	
Doses with symptoms (n, %)	2222 (42)	827 (18.6)	0.44 (0.41-0.48)
**Dosing related symptoms (number of dosing related reactions with symptom, % of doses)**			
Oropharyngeal	1537 (29)	696 (15.6)	0.54 (0.49-0.59)
Skin	169 (3)	22 (0.5)	0.15 (0.09-0.24)
Gastrointestinal	846 (16)	134 (3.0)	0.19 (0.16-0.23)
Lower respiratory tract	83 (2)	16 (0.4)	0.23 (0.12-0.39)

Abbreviations: OIT-oral immunotherapy; IRR- incidence rate ratio; CI- confidence interval

Table S5. Epinephrine use during the second year of the trial

Related to Treatment	Reaction	Modification of dosing
Yes	Subject dosed at home (500 mg) and developed pruritis, hives and shortness of breath. Family went to the emergency department where subject was treated with epinephrine, prednisone and famotidine. Family concern this was related to stress.	Took build-up BMOIT dose at home the following day (500 mg) prior to contacting us with no symptoms.
No	Medications used for treatment of allergic reaction to cashew includes epinephrine, bismuth subsalicylate, and diphenhydramine	None, Not related to BMOIT
No	Ate oatmeal that contained milk (accidental ingestion). Used epinephrine. Was on 50mg of BMOIT (build-up)	None, Not related to BMOIT
No	Rash treated with diphenhydramine, and then rash worsened, and c/o itching. School nurse administered epinephrine. Mom brought subject to emergency department for observation. BMOIT dose not taken that day.	None, Not related to BMOIT

Abbreviations: BMOIT- baked milk oral immunotherapy

Table S6. Detailed description of T cell populations selected for analysis

Flow Populations	Description	Rationale for initial selection	p-value*
Treg	CD4+CD127-CD25+; increase across BMOIT	Tregs are associated with tolerance	0.0104
CM+CD127-CD25+	Subset of antigen specific cells; increase across BMOIT	Population identified in Lewis et. al to be increased with CMA-BR	0.0002
CM+	Sorted population of CM+ cells (CD154+ and/or CD137+); decrease across BMOIT	Antigen specific population	0.0002
CM+NOT	Inverse population of CM+CD127-CD25+. Calculated as a %CD4 memory; decrease across BMOIT	Antigen specific population: Inverse population of CM+CD127-CD25+ from Lewis et. al.	<0.0001
CD154+CD137-	Sub-population from the CM+ sort gate; decrease across BMOIT	Antigen specific population	0.0415
CD154+CD137+	Sub-population from the CM+ sort gate; decrease across BMOIT	Antigen specific population	<0.0001
total CD154+	Sub-population from the CM+ sort gate; decrease across BMOIT	Antigen specific population	<0.0001
**scRNA-Seq Populations**			
CM+*FOXP3*+	CM+ cells expressing *FOXP3*; increase across BMOIT	Population identified in Lewis et. al to be increased in CMA compared to non-CMA	0.0009
CM+ Th2A	Based on gene module with >0.25 expression level/cell. Gene signature is based on previous studies describing pathogenic Th2/Tfh; decrease across BMOIT	Population identified in Lewis et. al to be increased in CMA compared to non-CMA	0.008
Ratio CM+FOXP3+/Th2A	Ratio of CM+FOXP3+/CM+Th2A populations described above; Increase over BMOIT	Ratio of populations identified in Lewis et. al	0.0033
CM+ C3	CM+ FOXP3+ Cluster 3 with high MHC II gene expression; increase across BMOIT	CM+ cluster population	0.0002
CM+ C5	CM+ FOXP3- Cluster 5 with Th1/Th17 gene expression; decrease across BMOIT	CM+ cluster population	0.0001
CM+ C4	CM+ FOXP3- Cluster 4 with CCR7 gene expression; decrease across BMOIT	CM+ cluster population	0.009
CM+ C10	CM+ FOXP3+ Cluster 10 with high CD137 and chemokine gene expression; no change across BMOIT	CM+ cluster population	0.708
CM+ C18	CM+ FOXP3+ Cluster 18 with interferon-responsive gene signature; no change across BMOIT	CM+ cluster population	0.562
CM+ C22	CM+ FOXP3- Cluster 22 with Th2 gene expression; no change across BMOIT	CM+ cluster population	0.838

*p-value significance calculated between Month 0 and 24 (paired t-test)

Table S7. Baked milk oral immunotherapy dosing schedule

**Dose #**	**Milk dose (mg) of milk protein**	**Study Phase**	**Comments**
1	0.1	Initial Dose Escalation	
2	0.2		
3	0.4		
4	0.8		
5	1.5		
6	3		Minimal starting dose
7	6	Initial Dose Escalation or build-up	
8	12		
9	25		
10	37.5	Build-up	
11	50		
12	75		
13	125		
14	200		
15	300		
16	500		
17	750		Minimum maintenance dose
18	1000		
19	1500		
20	2000	Maintenance	Goal Maintenance dose

Table S8. Dosing Schedule for Double-Blind, Placebo-Controlled Food Challenges

	**Baseline**		**Month-12 Blinded year of OIT**		**Month-24 Open-label OIT**		**Month-24 Open-label OIT**	
	Baked Milk		Baked Milk		Baked Milk		Unheated Milk	
Dose #	Milk Protein/ Placebo (mg)	Cumulative Dose (mg)	Milk Protein/ Placebo (mg)	Cumulative Dose (mg)	Milk Protein/ Placebo (mg)	Cumulative Dose (mg)	Milk Protein/ Placebo (mg)	Cumulative Dose (mg)
1	1	1	1	1	X	X	30	30
2	3	4	3	4	X	X	100	130
3	10	14	10	14	X	X	300	430
4	30	44	30	44	X	X	600	1030
5	100	144	100	144	X	X	1000	2030
6	300	444	300	444	444	444	1500	3530
7			600	1044	600	1044	2000	5530
8			1000	2044	1000	2044	2500	8030
9			2000	4044	2000	4044		

Doses listed are milligrams of milk protein or the equivalent amount of placebo powder.

Table S9. Detailed model parameters

Features	Outcome	Best Model	Hyper-parameters	Mean AUC (SD)	Cross-validation
Baseline	Baked Milk OFC	Random Forest	RandomForestClassifier(max_depth=1, max_features='log2', n_estimators=10, random_state=0)	0.647 (0.261)	kfold: n_splits=3, n_repeats=10
One Year Treatment	Baked Milk OFC	Elastic Net-Logistic	SGDClassifier(alpha=0.5, class_weight='balanced', l1_ratio=0.5, loss='log_loss', penalty='elasticnet', random_state=0)	0.552 (0.159)	kfold: n_splits=3, n_repeats=10
Baseline	Unheated Milk OFC	Ridge- Logistic	SGDClassifier(alpha=0.5, loss='log_loss', random_state=0)	0.621 (0.285)	kfold: n_splits=5, n_repeats=10
24 Months	Unheated Milk OFC	Ridge- Logistic	SGDClassifier(alpha=0.1, loss='log_loss', random_state=0)	0.806 (0.274)	kfold: n_splits=5, n_repeats=10

Abbreviations: AUC, area under the curve; SD, standard deviation; OFC, oral food challenge

Supplemental Figures

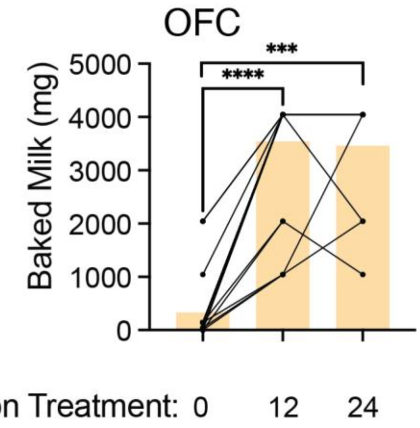

Figure S1. Maximum cumulative tolerated dose of baked milk by time on treatment. Baked milk oral food challenge outcomes indicated by maximum tolerated dose (mg) per subject grouped by time on treatment. Bars are median values. ***=<0.001, ****= <0.0001

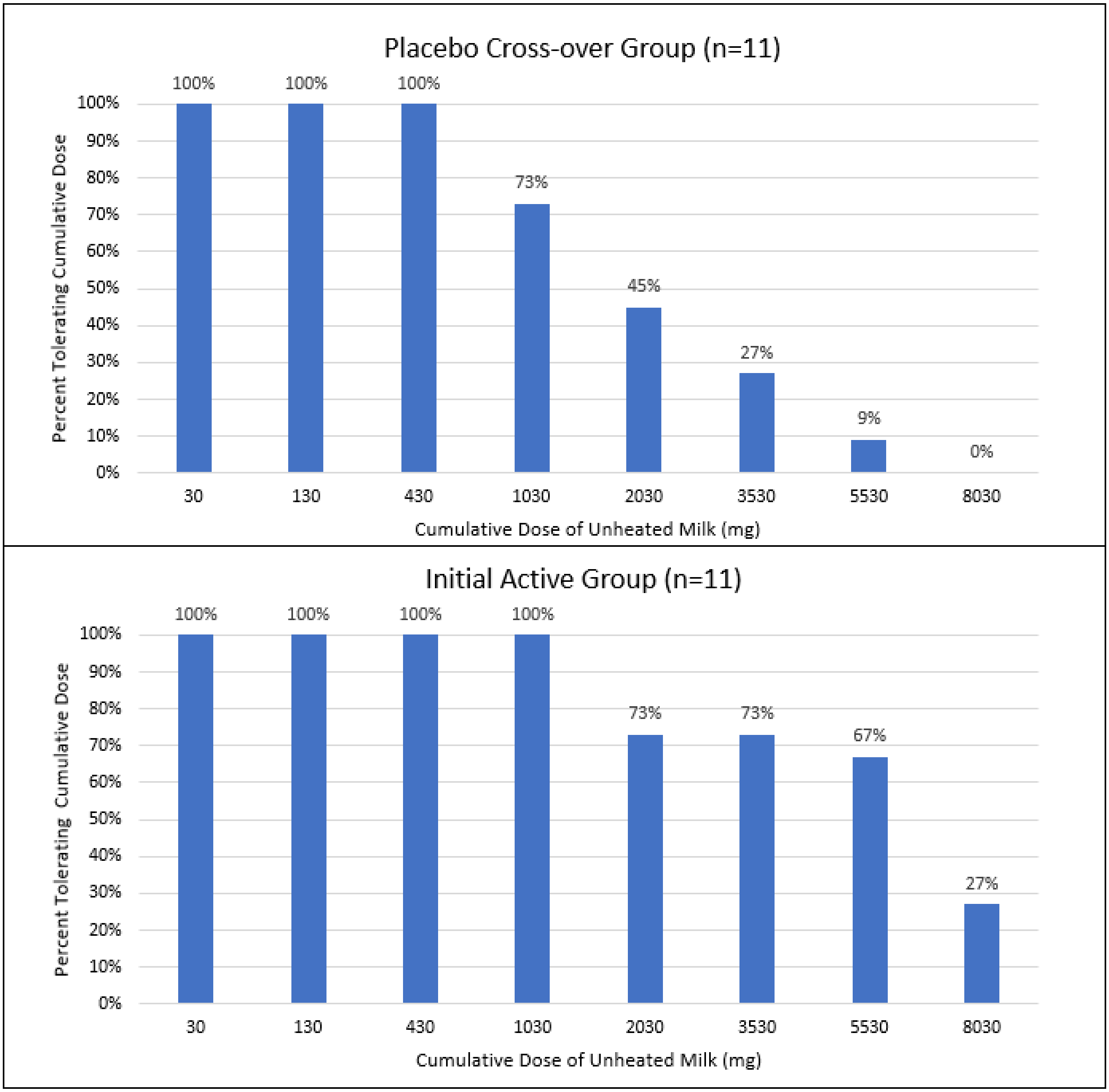

Figure S2. **Percent tolerating dose of unheated milk during month-24 DBPCFC**. Percent tolerating cumulative dose of unheated milk (mg of milk protein) during the month-24 DBPCFC, by group. Included those who completed the food challenge

Abbreviation: DBPCFC, double-blind placebo-controlled food challenge

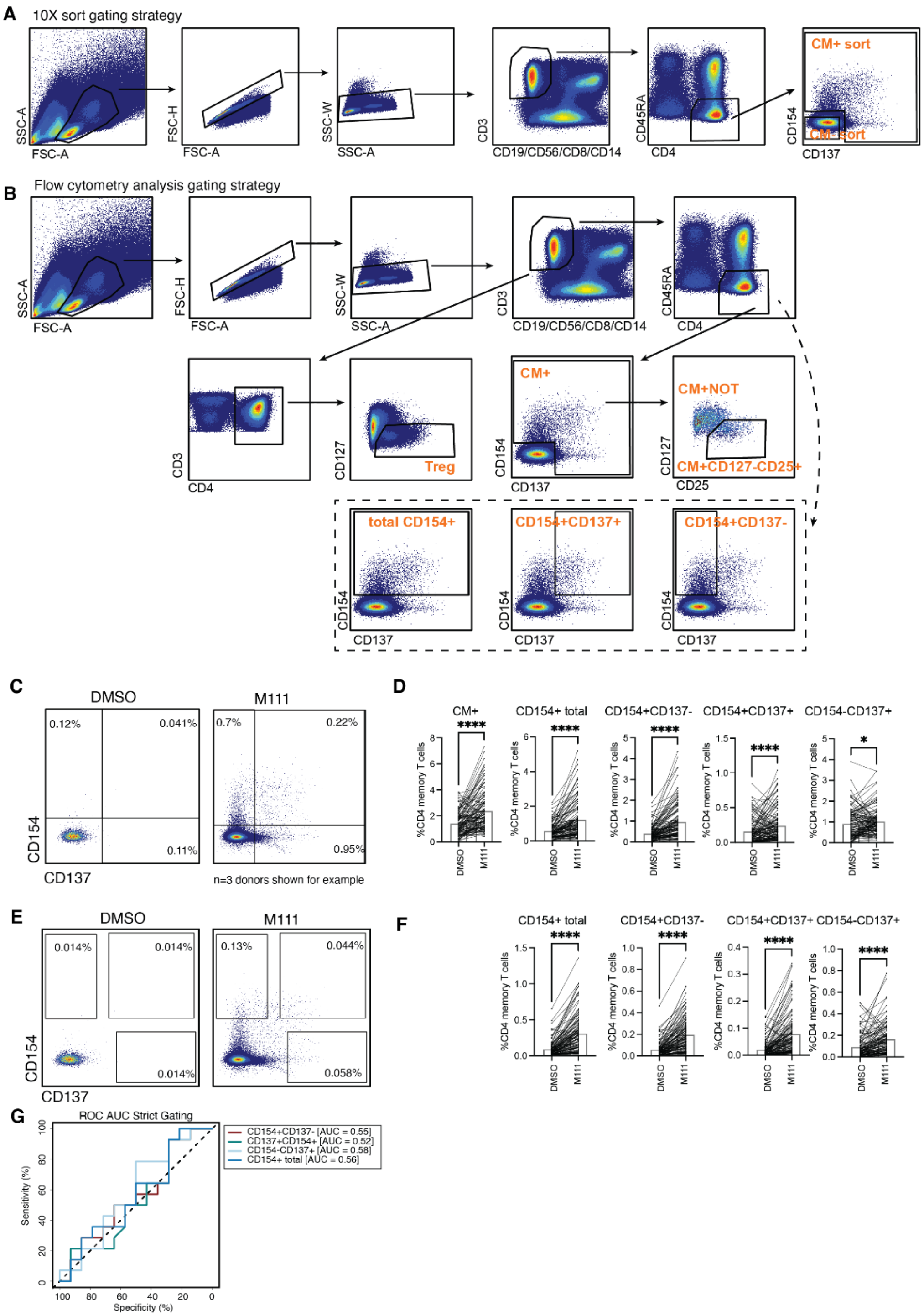

Figure S3. Sort and flow supplemental. **A)** Gating strategy for sort. **B)** Gating strategy flow cytometry analysis. **C)** DMSO/M111 examples following sort gating. **D)** Dot plots showing DMSO and M111 percentages in each indicated population. **E)** DMSO/M111 examples following strict gating. **F)** Dot plots showing DMSO and M111 percentages in each indicated population. **G)** ROC AUC analysis of strict gated populations. Statistical analysis was performed using paired Wilcoxan tests where p<0.0001 = **** and p<0.05 = *.

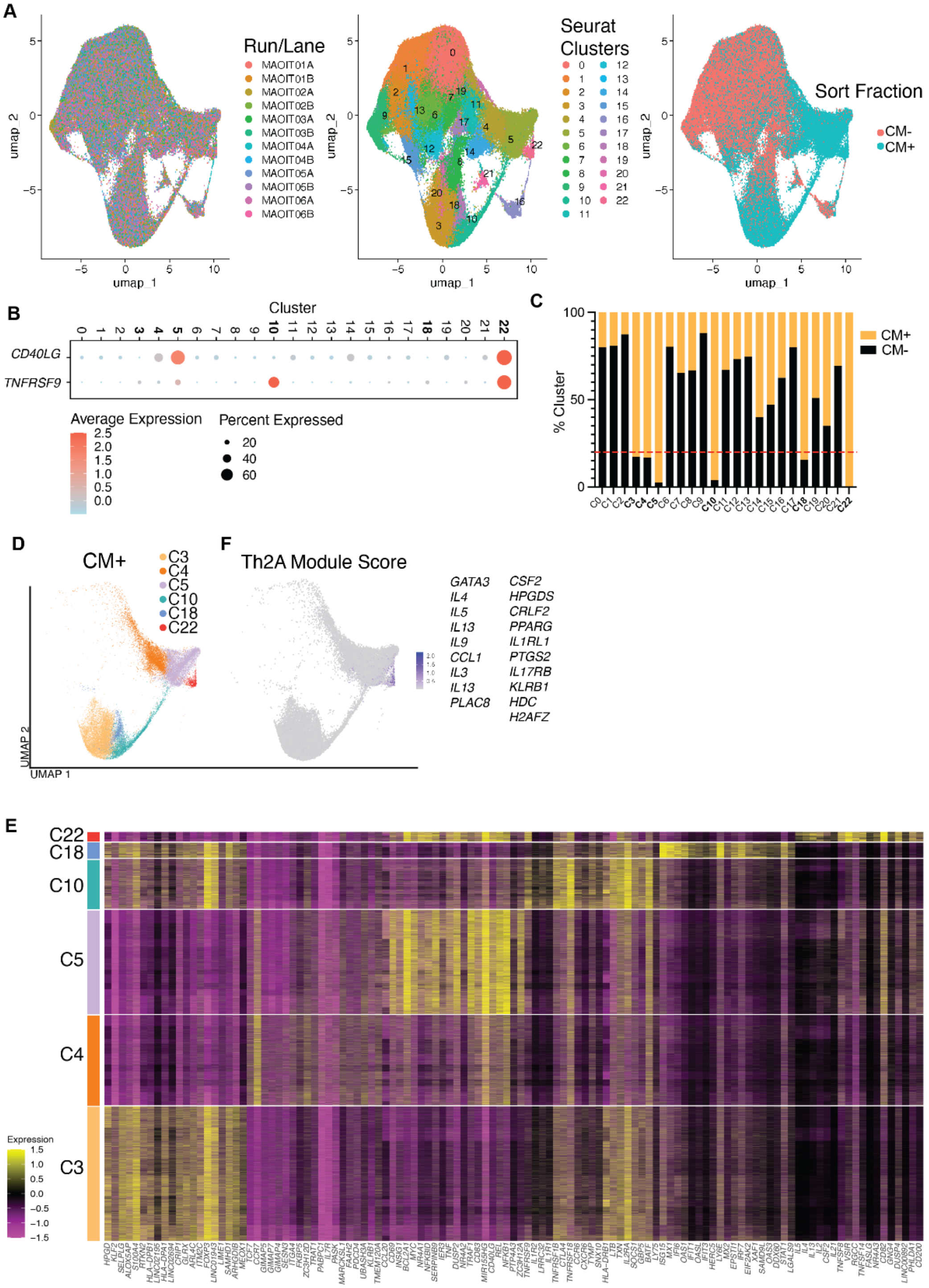

Figure S4. scRNA-Seq supplemental. A) UMAP plots showing run lane (left), Seurat clusters (middle), and antigen specific sort fraction (right). **B)** Dot plot showing RNA experssion of sort markers across all clusters. **C)** Bar plot showing percentage of cells in each cluster labeled by sort fraction. **D)** UMAP of selected CM+ clusters. **E)** Heatmap of highly expressed genes in each CM+ cluster. **F**) UMAP of selected CM+ clusters colored by pathogenic Th2 module score.

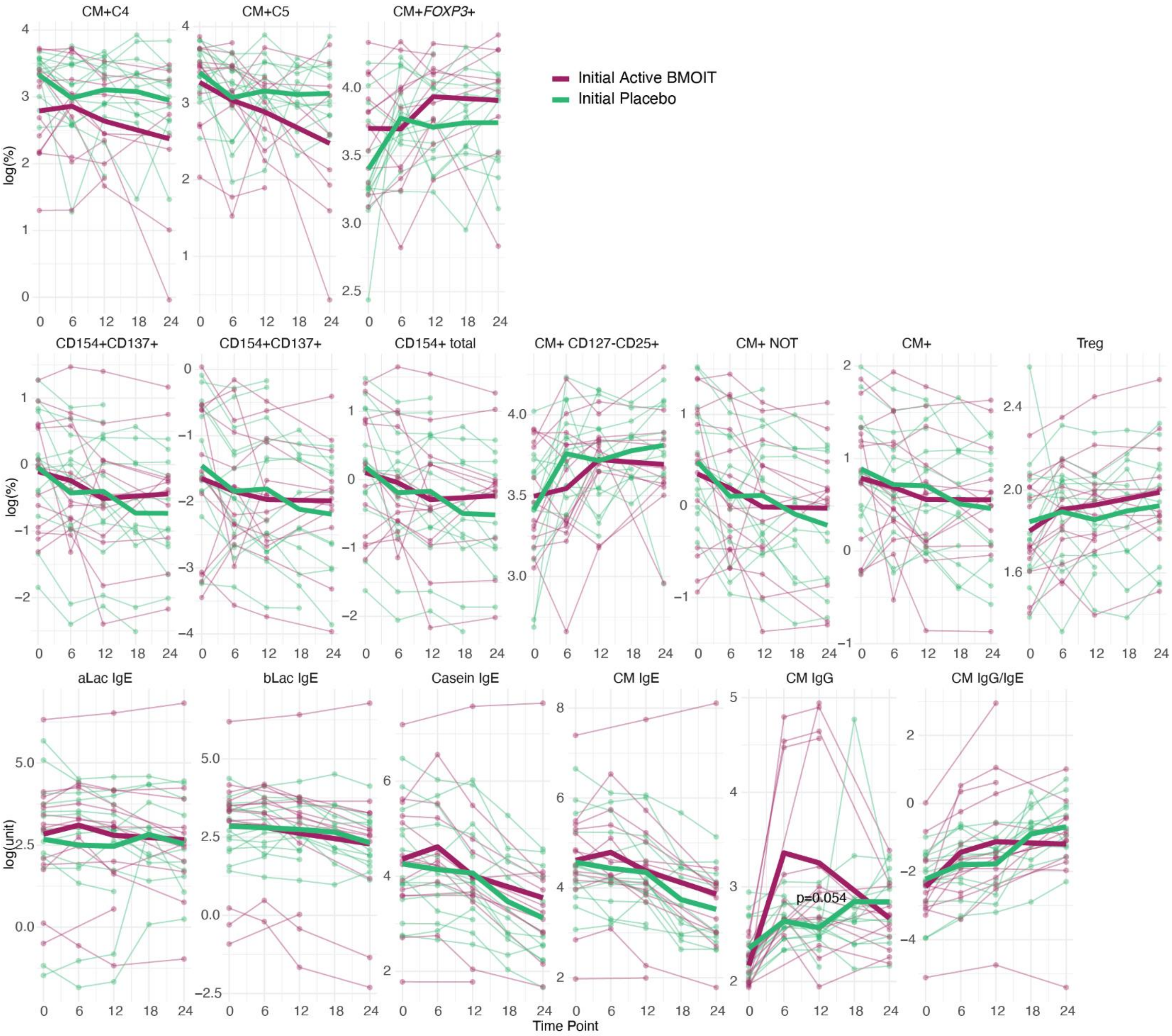

Figure S5. T cell and antibody measurements by treatment group across all time point. Line plots showing all T cell populations and antibody measurements across all timepoints colored by treatment group. Lines connect each subject and the bolded lines are the means of that group.

Abbreviations: BMOIT, baked milk oral immunotherapy

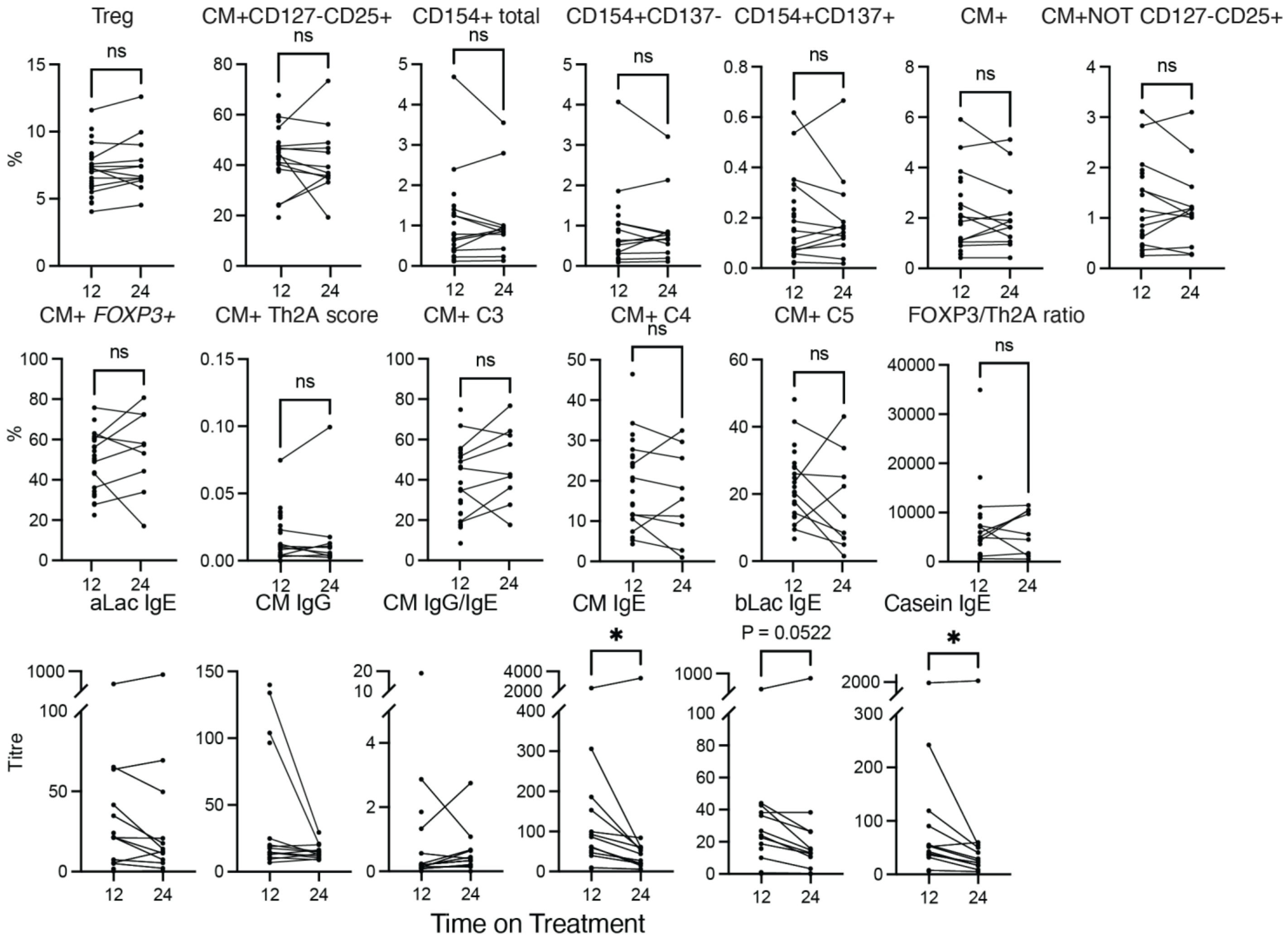

Figure S6. Population changes from 12 to 24 months on treatment. Line plots comparing 12 to 24 month timepoints for each T cell population and antibody measurement. Subjects with data at both timepoints are connected with a line. Statistics were run by both paired and unpaired t-test. Significances noted here are paired analysis.

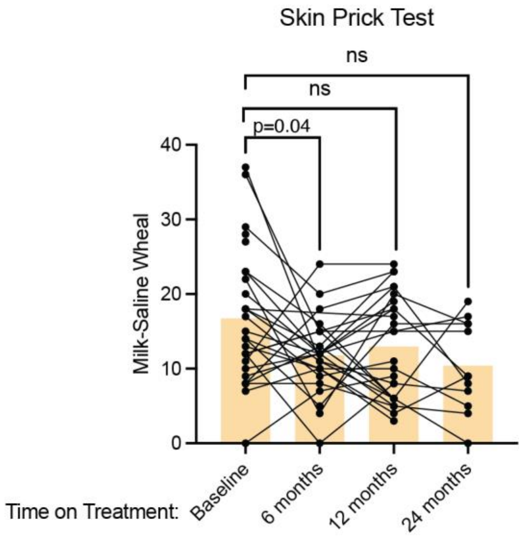

Figure S7. **Skin prick test**. Milk skin prick test results reported as Milk minus Saline Wheal measurement and grouped by time on treatment.

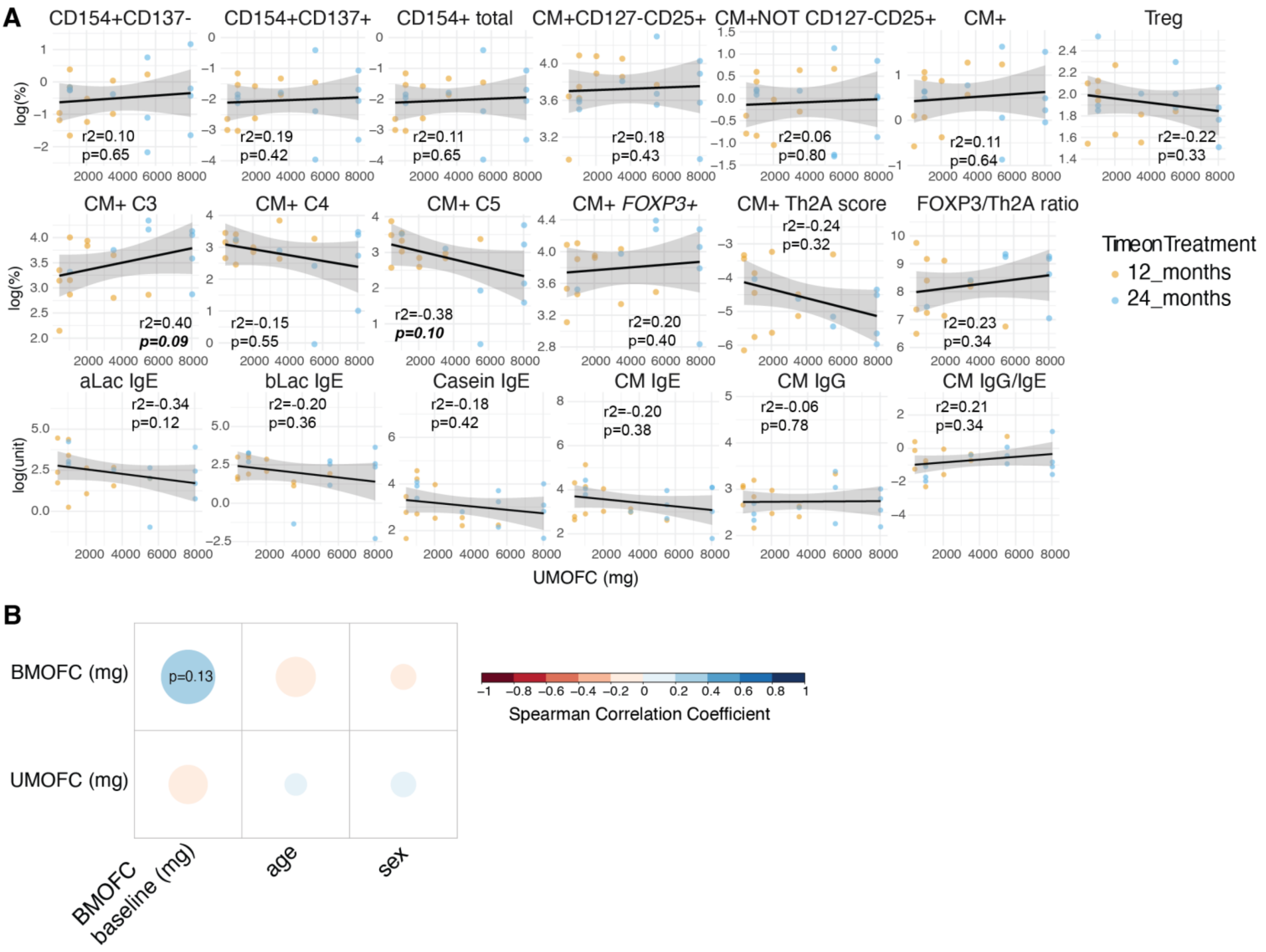

Figure S8. Correlation analysis supplemental. A) Scatter plots showing correlations of antibody measurements, scRNA-Seq populations, and flow cytometry populations with UMOFC doses (mg). Color of the dot represents treatment timepoint. Significance of correlations are noted on the plots. **B)** Correlations of BMOFC and UMOFC outcomes (mg) with clinical features where color and size of the dot represent spearman correlation coefficients.

Supplemental Appendices

Appendix 1. Full inclusion/exclusion Criteria

1. *Inclusion/Exclusion Criteria*

Patients who meet *all* the following criteria are eligible for enrollment as study participants, including participants who:

- Are age 3-18 years, male or female, any ethnicity or race
- Provide signed informed consent by parent or legal guardian and informed assent if applicable
- Have a history of symptomatic reactivity to cow's milk (i.e. eczema, urticarial, upper or lower respiratory symptoms, GI disturbances, rash, oral symptoms)
- Have a skin prick test positive to milk (diameter of wheal 3 mm ≥ negative control) and serum milk-specific IgE level >5 kU/L within the past 6-12 months
- Have a positive reaction to a cumulative dose of ≤444 mg of baked milk protein in the initial qualifying DBPCFC.
- Use an effective method of contraception by females of childbearing potential to prevent pregnancy and agree to continue to practice an acceptable method of contraception for the duration of their participation in the study.
- Have self-injectable epinephrine available at all times

Patients who meet *any* of these criteria are not eligible for enrollment as study participants, including participants who:

- Have a history of severe anaphylaxis resulting in hypotension, neurological compromise, or mechanical ventilation
- Have a history of intubation related to asthma
- Tolerate more than 444 mg of baked milk protein at the initial qualifying DBPCFC.
- Allergy to placebo ingredients OR reacts to any dose of placebo during the qualifying OFC.

- Poor control of atopic dermatitis
- Are unable to tolerate at least 3 mg of baked milk protein on dose escalation day
- Are pregnant or lactating
- Have severe asthma defined by 2007 NHLBI Criteria Steps 5 or 6
- Have severe or poorly controlled asthma defined by with any of the following criteria:
    1. FEV1<80% of predicted
    2. ICS dosing of >500 mcg daily of fluticasone (or equivalent inhaled corticosteroids based on NHLBI dosing chart) or
    3. ≥ 1 hospitalization in the past year for asthma or
    4. > 1 ER visit in the past 6 months for asthma
- Use of steroid medications (oral steroids, such as prednisone or Medrol, steroid injections, such as Kenalog, or IV or oral corticosteroid burst) in the following manners: History of daily oral steroid dosing within 4 weeks prior to baseline visit *or* for > 1 month during the past year *or* >2 burst oral steroid courses in the past 6 months.
- Are unable to discontinue antihistamines for 5 days for long acting and 3 days for short acting prior to skin testing or food challenges
- Are receiving omalizumab, mepolizumab, beta- blocker, ACE inhibitor, angiotensin-receptor blockers, calcium channel blockers, or tricyclic antidepressant therapy
- Have used immunomodulatory therapy (not including corticosteroids) or biologic therapy within the past year
- Have participated in any interventional study for treatment of a food allergy in the past 6 months
- Are on 'build up phase' of environmental allergen immunotherapy. Subjects tolerating maintenance allergen immunotherapy can be enrolled.
- Have a history of eosinophilic esophagitis in the past 3 years
- Have a chronic disease (other than asthma, atopic dermatitis, rhinitis) requiring therapy (e.g., heart disease, diabetes)

- Have used an investigational drug within 90 days or plan to use an investigational drug during the study period
- Severe reaction at initial DBPCFC, defined as:
    - Life-threatening anaphylaxis
    - Requiring overnight hospitalization

Appendix 2. Study Product Details

The milk powder (organic, nonfat dry milk powder) was purchased by the University of North Carolina (UNC) from Milky Whey, Inc. & TMW International. UNC analyzed the milk protein content in each lot and performed SDS-PAGE, densitometry analysis, and bioburden testing. The placebo, irradiated tapioca flour was purchased by UNC from Ener-G Foods Inc. UNC analyzed each lot for the absence of milk protein and performed SDS-PAGE, densitometry analysis, and bioburden testing. UNC provided individualized packaged doses for dispensing for home dosing and bulk product for use by JHH nutritionist. Tapioca flour was selected due to similarity in appearance.

Participants were given instructions on how to prepare, store, and administer the OIT dose at home. The OIT powder was stored in the refrigerator. To prepare the dose, the family was instructed to prepare a cupcake or muffin batter using any preferred, dairy-free recipe that was also free from their child's other allergens. They then poured the batter into a regular size muffin tray. Then, added 1 pre-measured OIT powder dose to each individual muffin tin and stirred well. The cupcake or muffin was then required to be baked at 350°F for at least 30 minutes. The child was instructed to ingest one cupcake or muffin each day. Instructions were given for avoiding strenuous exercise for at least 2 hours after taking their dose. Participants were advised to contact the study team if the child missed a dose or if they were ill.

All doses given during a food challenge or in clinic were prepared according to our Standard of Operation for Oral Food Challenge Preparation with pre-specified cake recipes developed by the Johns Hopkins Research Nutrition Team.

Appendix 3. Double-blind, placebo-controlled food challenge details

The baseline baked milk food challenge was performed as a DBPCFC with the active portion consisting of cake with 444mg of baked milk protein. The cake was administered over six steps (1mg, 3mg, 10mg, 30mg, 100mg, and 300mg milk protein). For the placebo portion of the DBPCFC, tapioca flour was substituted for milk in the cake recipe. The cake was prepared using pre-specified recipes developed by the JH Research Nutrition Team. The month 12 baked milk DBPCFC had a cumulative dose of 4044mg (1mg, 3mg, 10mg, 30mg, 100mg, 300mg, 600mg, 1000mg, and 2000mg milk protein). The month 24 baked milk DBPCFC had a cumulative dose of 4044mg, but a higher starting dose since all participants were known to be on active treatment (444mg, 600mg, 1000mg, 2000mg milk protein). The month 24 unheated milk challenge had a maximum cumulative dose of 8030mg (30mg, 100mg, 300mg, 600mg, 1000mg, 1500mg, 2000mg, 2500mg). MTD was defined as the maximum cumulative dose of milk protein ingested without dose-limiting symptoms.

Appendix 4. Sample Size, Randomization, and Blinding

Sample Size

The sample size was determined based on 1) data from previous studies and 2) hypothesis testing for test of difference in proportions. Based on prior studies, we anticipated that at most 10% of subjects in the placebo arm and at least 60% of subjects in the treatment arm would tolerate 4 grams of baked milk at the month-12 DBPCFC. To achieve an alpha of 0.05 and a power of 0.8, 14 subjects were needed in each group. We choose 15 participants per group to allow for a 7% drop out rate.

Randomization

Patients were randomized with a one-to-one allocation of treatment to placebo using a block randomization scheme. Patients were enrolled by a study nurse or clinician. When the patient had been deemed eligible for randomization, a study nurse or clinician communicated the need for randomization to the research pharmacist using a "Randomization Request" form. The research pharmacist randomized the patient using the random code. The random code was generated using computer generated sheets with block stratified assignment of block size of six, 1:1 distribution of active to placebo. The participants were randomized using the next available slot on the random code, which indicated whether the participant was randomized to active or to placebo.

After randomization, the research pharmacist recorded the patient's assignment in the randomization log. They also shared a copy of the patient's randomization assignment with the nutritionist. The research pharmacist sent confirmation of randomization (but not randomization assignment) to the study nurse or clinician.

Blinding

Patients, study coordinators, nurses, and clinicians were blinded to treatment arm assignment. The pharmacist and nutritionist remained un-blinded and had access to the randomization log. The pharmacist dispensed the patient's home dosing supplies upon request by the clinician. The investigational product was labeled with a blinded label and an unblinded tear-off label indicating whether the product was active or placebo. The unblinded tear-off label was removed by the unblinded pharmacy staff prior to dispensing. The nutritionist prepared and dispensed the appropriate dose for

the subject to take in the PCRU (during the oral food challenges, initial dose escalation day, and up-dosing) once a request had been made by the clinician. The in-office dosing kits were labeled with a blinded label. Thus, the study clinicians and patients remained blinded to treatment arm assignment.

Appendix 5. Impact of COVID-19 pandemic

Due to the COVID-19 pandemic, in-person research visits were stopped from March 2020 until July 2020. Those on build-up remained on their current dose and those in maintenance continued their 2000 mg dose. Participants were shipped investigational product during this time with virtual visits performed every 2 weeks to 2 months depending on treatment phase. In summary, 17 participants (8- initial BMOIT group, 9-placebo cross-over group) were in year 2 at this time with 6 having additional time in build-up, 2 with additional time in maintenance, and 1 delaying their cross-over IDE.

Full protocol can be accessed by contacting the authors and is available on clinicaltrials.gov.
